# Integrating a crop growth model and radiative transfer model to improve estimation of crop traits based on deep learning

**DOI:** 10.1093/jxb/erac291

**Published:** 2022-06-30

**Authors:** Qiaomin Chen, Bangyou Zheng, Tong Chen, Scott C Chapman

**Affiliations:** School of Agriculture and Food Sciences, The University of Queensland, St Lucia, 4067, QLD, Australia; CSIRO Agriculture and Food, Queensland Biosciences Precinct 306 Carmody Road, St Lucia, 4067, QLD, Australia; CSIRO Agriculture and Food, Queensland Biosciences Precinct 306 Carmody Road, St Lucia, 4067, QLD, Australia; School of Information Technology and Electrical Engineering, The University of Queensland, St Lucia, 4067, QLD, Australia; School of Agriculture and Food Sciences, The University of Queensland, St Lucia, 4067, QLD, Australia; CSIRO Agriculture and Food, Australia

**Keywords:** APSIM, biological constraints, canopy and leaf biophysical traits, canopy reflectance, PROSAIL, unmanned aerial vehicle

## Abstract

A major challenge for the estimation of crop traits (biophysical variables) from canopy reflectance is the creation of a high-quality training dataset. To address this problem, this research investigated a conceptual framework by integrating a crop growth model with a radiative transfer model to introduce biological constraints in a synthetic training dataset. In addition to the comparison of two datasets without and with biological constraints, we also investigated the effects of observation geometry, retrieval method, and wavelength range on estimation accuracy of four crop traits (leaf area index, leaf chlorophyll content, leaf dry matter, and leaf water content) of wheat. The theoretical analysis demonstrated potential advantages of adding biological constraints in synthetic training datasets as well as the capability of deep learning. Additionally, the predictive models were validated on real unmanned aerial vehicle-based multispectral images collected from wheat plots contrasting in canopy structure. The predictive model trained over a synthetic dataset with biological constraints enabled the prediction of leaf water content from using wavelengths in the visible to near infrared range based on the correlations between crop traits. Our findings presented the potential of the proposed conceptual framework in simultaneously retrieving multiple crop traits from canopy reflectance for applications in precision agriculture and plant breeding.

## Teaser

We proposed and validated a conceptual framework to introduce biological constraints in a synthetic dataset used for training a deep learning model to improve estimation of crop traits.

## Introduction

Since the 1960s, satellite imagery to measure reflectance of the Earth’s surface at a scale of tens of metres has been used to monitor vegetation health and estimate and forecast changes in vegetation cover (see related reviews, e.g. Dorigo *et al.*, 2007; Baret and Buis, 2008; Thenkabail *et al.*, 2019). More recently, these imagery methods have been deployed in proximal sensors (e.g. planes, drones, and vehicles) to analyse vegetation at higher resolutions (i.e. sub-centimetre scales) in a research domain sometimes referred as ‘high-throughput phenotyping’ (e.g. Fahlgren *et al.*, 2015; Chapman *et al.*, 2018).Nanosatellites with higher temporal (revisit time down to 1 d), spatial (0.3–1.0 m), and spectral (narrower hyperspectral bandwidth, 1–10 nm) resolutions are also being deployed (e.g. McCabe *et al.*, 2016; Sozzi *et al.*, 2018). Since sensors and imaging technologies can rapidly measure multiple crop traits across time and space in a cost- and labour-efficient way, they present potential to benefit applications in precision agriculture and in plot experiments conducted in plant breeding and agronomy.

Depending on the electromagnetic spectrum used in sensors or cameras, different crop traits can be retrieved from sensing data. The visible range at ~400–750 nm (from RGB cameras) can be used to measure the morphological, geometric, and colour properties, such as ground cover (e.g. Duan *et al.*, 2017; Wan *et al.*, 2021), ear/head number, or density (e.g. Madec *et al.*, 2019; David *et al.*, 2021). From spectral cameras, narrow bands in the visible range from 400 nm to 700 nm can also be used to measure some plant biochemical properties such as pigment content (e.g. chlorophyll, carotenoid, and anthocyanins) (e.g. Ustin *et al.*, 2009) and nitrogen content (e.g. Jay *et al.*, 2017). The visible to near infrared (NIR) range at ~400–1100 nm is generally used to estimate the fractional ground cover of vegetation and the leaf area index (LAI) (e.g. Hu *et al.*, 2021). The shortwave infrared range at ~1200–2500 nm is suitable to measure plant water content (e.g. Jin *et al.*, 2013). The longwave infrared range (sometimes called thermal infrared) at ~7.5–13 µm can be used to measure plant temperature and is usually proposed for detection of water-related or heat-related stresses (e.g. Zhang *et al.*, 2019). Based on the estimation of variables associated with stress status, sensing data have been used for abiotic/biotic stress detection and disease diagnoses in crop protection (e.g. Mahlein *et al.*, 2012).

The retrieval of crop traits from spectral signals (canopy reflectance and its derived variables such as vegetation index) is based on the structural and biochemical relationships between traits and spectra. These can directly come from the cause–effect relationship expressed in radiative transfer models (RTMs) and be learned from training data collected from field experiments (experimental data) or generated by RTMs (synthetic data). Compared with experimental data, the main advantage of synthetic data is to allow construction of a training dataset to represent the diverse range of situations generated by interactions of genotype, environment, and management, as well as observation conditions (Dorigo *et al.*, 2007; Baret and Buis, 2008), and provides a capability to generalize the prediction of crop traits in field conditions. There is an increasing interest in the application of RTMs in crop trait retrieval studies, in which PROSAIL is a popular model and has been widely used for variable retrieval, as reviewed by Berger *et al.* (2018).

For those retrieval studies based on the inversion of the cause–effect relationship between crop traits and reflectance expressed in RTMs, numerical optimization and look-up table (LUT) are the two major retrieval methods. Numerical optimization is realized by comparing the observed and simulated spectral signal to find the optimal solution (e.g. Danner *et al.*, 2019; Lunagaria and Patel, 2019), while LUT is achieved by searching the entire LUT for optimal solutions stored in the LUT (e.g. Quan *et al.*, 2017; X.Q. Xu *et al.*, 2019; Zhu *et al.*, 2019). Machine learning methods including neural networks are also increasingly used to rebuild (learn) the inverse relationship between reflectance and crop traits (e.g. Bacour *et al.*, 2006; Upreti *et al.*, 2019). As summarized in the literature, different methods have advantages and limitations, with no obvious global solution, but machine learning methods are assumed to be superior in representing relationships between model input and output from training data, and are more computationally efficient once the model is fully trained (e.g. Dorigo *et al.*, 2007; Verrelst *et al.*, 2015).

A major challenge for synthetic data approaches is the ‘ill-posed’ problem to retrieve crop traits from sensing data (e.g. Baret and Buis, 2008; Jacquemoud *et al.*, 2009). This problem is caused by the model uncertainty which results from its simplification of the canopy structure and biochemistry, so that more than one state situation of a canopy could result in exactly the same reflectance (e.g. Jacquemoud *et al.*, 1995; Combal *et al.*, 2003) However, in practical applications, this problem is caused by the local optimization of a criterion (i.e. objective function, cost function, or loss function) that can be obtained from different solutions (parameter combinations). In synthetic data, this problem is aggravated by the poor selection of combinations of input parameter values; that is, not accounting for biophysical limitations among combinations of parameter values that actually exist in the real-world. Without consideration of these biophysical constraints among parameters, the ill-posed nature of the inverse problem results in multiple solutions that may be spread across the whole parameter space rather than centred around the true solution (e.g. Atzberger, 2004).

The ill-posed problem can be partly alleviated by using prior knowledge to strengthen constraints on individual input parameters or among parameters. The simplest and most common method is to define the appropriate range of parameters with experimental data, which have been proved to improve estimation accuracy (e.g. Li *et al.*, 2019). In inversion studies with LUT, using the medium or mean of the best *k* (*k*>1) solutions is another common way to alleviate the ill-posed problem (e.g. Combal *et al.*, 2003; Quan *et al.*, 2017). Studies also provided evidence for using additional biological or spatial constraints to improve estimation accuracy: for example, M. Xu *et al.* (2019) introduced a matrix-based LUT consisting of two vegetation indexes (one sensitive to leaf chlorophyll and the other sensitive to LAI) to consider the effect of LAI on leaf chlorophyll content estimation; X.Q. Xu *et al.* (2019) developed a LUT of maximum conditional probabilities of LAI and canopy chlorophyll content based on a Bayesian network for trait estimation; and Atzberger (2004) proposed an object-based method to introduce the spatial constraints of the target trait (LAI, leaf chlorophyll content, and leaf water content) to improve its estimation accuracy. In addition, multiangular observations (e.g. Jay *et al.*, 2017; Roosjen *et al.*, 2018) were also investigated to address the ill-posed problem.

An alternative approach is to link a crop growth model (CGM) to RTM, which provides a more straightforward solution to address the ill-posed problem by directly constraining the combinations of RTM input parameters with crop physiology algorithms that are built into the CGM. Theoretically, the integration can be implemented in two ways by (i) calibrating/parameterizing the CGM using canopy reflectance and then predicting target crop traits using the calibrated CGM (Method 1); or (ii) converting CGM output variables into RTM input parameters and then applying retrieval methods (e.g. LUT) on these constrained crop traits and corresponding canopy reflectance (Method 2). Method 1 can directly retrieve those traits not included in RTM inputs such as crop yield and also provide estimation of traits across the whole growth season by running calibrated CGMs (e.g. Thorp *et al.*, 2012; Machwitz *et al.*, 2014; Guo *et al.*, 2019). Method 2 provides a more direct way to predict target traits included in RTM inputs from reflectance data without additional needs of weather and management data used in CGMs, which is more applicable in practice but has been rarely discussed. In this study, for the first time, we implemented Method 2 to link a CGM and RTM for retrieving crop traits from canopy reflectance.

Previous studies have indicated that radiometric information is not sufficient to accurately estimate crop traits, and some prior information is needed to improve the estimation accuracy (e.g. Combal *et al.*, 2003). The aim of this study is to compare synthetic datasets with and without biological constraints to retrieve four crop traits (i.e. LAI, leaf chlorophyll content, leaf dry matter, and leaf water content) of wheat. The biological constrains were introduced through integrating a CGM (e.g. Agricultural Production System Simulator, APSIM) and an RTM (e.g. PROSAIL) in the way described in Method 2, so that PROSAIL input parameters are constrained to APSIM biology. We further investigated the effects of observation geometries, wavelength ranges, and retrieval methods on estimation accuracy of the four crop traits.

## Materials and methods

### Overview

**Fig. 1.**
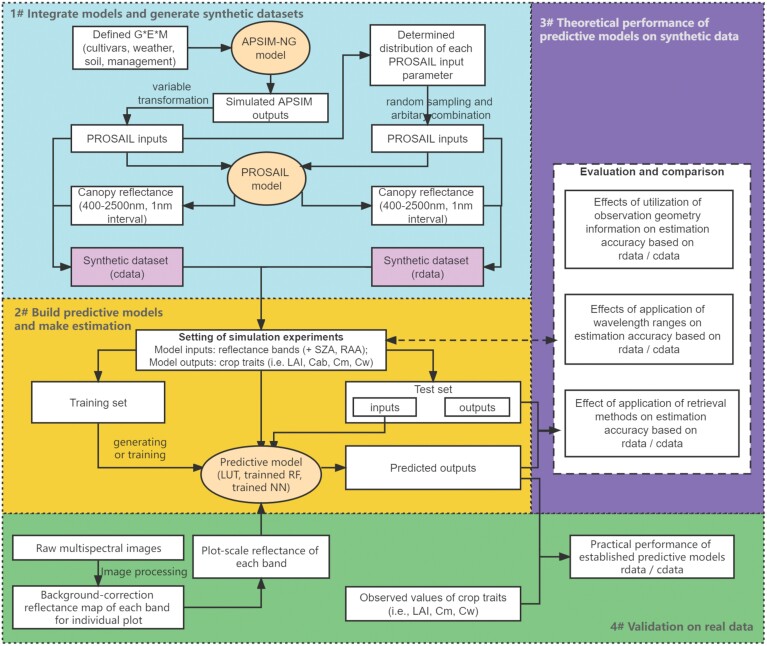
Research flow map. APSIM-NG denotes Agricultural Production Systems sIMulator (APSIM) Next Generation, which is a crop model. PROSAIL is a radiative transfer model, coupling a leaf optical property model (PROSPECT-D) and a canopy bidirectional reflectance model (4SAIL). Observation geometry information: SZA, solar zenith angle; RAA, relative azimuth angle. Crop traits: LAI, leaf area index; Cab, leaf chlorophyll content per leaf area; Cm, leaf dry matter content per leaf area; Cw, leaf water content per leaf area. Predictive model (or retrieval method): LUT, look-up table; RF, random forest; NN, feedforward neural network. Synthetic dataset: cdata, with biological constraints; rdata, without biological constraints.

This research consists of several key steps (Fig. 1), summarized as follows. APSIM and PROSAIL models were integrated by passing variables from the former to the latter based on variable transformation relationships. A set of defined conditions for wheat growing (characterized by genotype, environment, and management) and observation conditions (determined by local latitude, day of year, and time of day) was set up to run APSIM and PROSAIL for simulation of crop traits and canopy reflectance, which resulted in two types of synthetic datasets. The concept of a ‘synthetic dataset’ is relative to a real dataset collected from practical environments (e.g. field experiments). Here the ‘synthetic dataset’ was generated by physical models including APSIM and PROSAIL. According to research objectives, these synthetic datasets were reconstructed and used in a series of analyses defined below to investigate the effects of application of observation geometry, wavelength range, and retrieval methods on estimation accuracy of target crop traits. Finally, the prediction models were applied to real multispectral images collected in wheat plots contrasting in canopy structure, to evaluate the practical performance of this ‘synthetic data-derived’ predictive model.

### Models and their integration

APSIM Next Generation (https://www.apsim.info/apsim-next-generation/) is the new version of APSIM, which is an evolution of the classic version 7.10 (Holzworth *et al.*, 2018). APSIM Next Generation or APSIM is driven by major processes in crop physiology and interactions with environment factors and management practices, and is widely used to simulate crop development and growth (e.g. LAI or dry weight of organ parts) during the growth season at a daily time step. APSIM has been validated in many regions around the world for wheat (e.g. Chenu *et al.*, 2017). APSIM is not a structural model, so the crop variables such as biomass and leaf area per unit ground area are state variables computed each day as single variables, and do not have any 2D or 3D structural attributes. The model does utilize geometrical theory to determine how the relationship between LAI and light extinction coefficient (a crop trait) affects interception of incoming radiation each day, including the effects of plant row spacing, but APSIM Next Generation does not account for direct versus diffuse light conditions or change in radiation over the day (Holzworth *et al.*, 2018).

PROSAIL is the combination of PROSPECT (a leaf optical property model) and SAIL (a canopy bidirectional reflectance model). PROSAIL links the spectral variation of canopy reflectance (mainly related to leaf biochemical contents) with its directional variation (primarily related to canopy architecture and soil/vegetation contrast), which is key to simultaneously estimating canopy biophysical variables such as leaf chlorophyll content and LAI (Jacquemoud *et al.*, 2009). The current version of the PROSAIL model, PROSAIL-D, is available for download (http://teledetection.ipgp.jussieu.fr/prosail/, retrieved on 18 July 2022). Input and output variables of this PROSAIL model are presented in Table 1. The 14 input parameters can be divided into four categories: leaf properties [leaf mesophyll structure parameter (Ns), leaf water content per leaf area or leaf equivalent water thickness (Cw), leaf dry matter content per leaf area (Cm), leaf Chl *a* and *b* content per leaf area (Cab), leaf carotenoid content per leaf area (Car), leaf anthocyanin content per leaf area (Cant), and leaf brown pigment concentration (Cbrown)], background soil properties (rsoil), canopy architecture [LAI, leaf inclination distribution function (LIDF), and hot spot size parameter (hspot)], and solar-object-sensor observation geometry [solar zenith angle (SZA), viewing zenith angle (VZA), and relative azimuth angel (RAA)]. PROSAIL can output directional canopy reflectance, which is also represented using canopy reflectance or reflectance without explicit specification in the following sections. For further details, refer to the original papers about the PROSPECT (Jacquemoud and Baret, 1990) and SAIL model (Verhoef, 1984).

**Table 1:** Description of input parameters and output of PROSAIL

Variable	Unit	Description
Input		
Ns	Unitless	Leaf mesophyll structure parameter, relates to the cellular arrangement within the leaf.
Cw	g cm^−2^ or cm	Leaf water content (g cm^−2^) or leaf equivalent water thickness (cm)
Cm	g cm^−2^	Leaf dry matter content per leaf area
Cab	µg cm^−2^	Leaf Chl *a* and *b* content per leaf area
Car	µg cm^−2^	Leaf carotenoid content per leaf area
Cant	µg cm^−2^	Leaf anthocyanin content per leaf area
Cbrown	Unitless	Leaf brown pigment concentration
rsoil	Unitless	Reflectance of soil as a libertarian surface. It is usually adjusted by a soil brightness factor: asoil (to be multiplied with single rsoil spectrum) or psoil (scaling factor between the two model-implemented rsoil spectra—wet versus dry).
LAI	m^2^ m^−2^	Leaf area index
LIDF	–	Leaf inclination distribution function. There are two methods provided to calculate LIDF: use two parameters (LIDFa and LIDFb) or a single parameter (ALA for average leaf angle, degree)
hspot	m m^−1^	Hot spot size parameter. It is primarily designed to correct the canopy reflection regarding bidirectional effects. Hot spot effect is the case where a spot displays maximum reflectivity and appears brighter than surroundings because of no visible shadows at the hot spot position where the sensor is in direct alignment between the sun and the ground target.
SZA	°	Solar zenith angle (from vertical)
VZA	°	Viewing (or observing) zenith angle (from vertical)
RAA	°	Relative azimuth angle. It is equal to viewing azimuth angle minus solar azimuth angle.
Output		
resv	Unitless	Directional canopy reflectance

The coupling of APSIM and PROSAIL is realized by passing output variables of APSIM to PROSAIL as input variables. This permits the coupling model to estimate canopy reflectance from 400 nm to 2500 nm in 1 nm intervals at defined observation conditions (determined by latitude, day of year, and time of day) given that required parameters are specified. The transformation of variables is based on a series of equations (Table 2) and details are provided in Supplementary Protocol S1.

**Table 2:** Variable transformation from APSIM output to PROSAIL input

APSIM output variable	Transformation formula	PROSAIL input variable
LAI_Total_, LDW	Ns=(0.9×SLA+0.025)/(SLA−0.1) (Jacquemoud and Baret, 1990) where SLA=10×LAI_Total_/LDW	Ns
Z_s_, LAI_Total_, LAI_Dead_	$C_{w}=\left\{ \begin{array}{ll} -0.000196\;\;\;Z_{s}+0.0298 & \left(f_{dead}=0\right)\\ 0.0223\;\;\;exp\left(-1.90\;\;\;f_{dead}\right) & \left(f_{dead}>0\right) \end{array}\right.$ where f_dead_=LAI_Dead_/LAI_Total_	Cw
LDW, LAI_Total_	Cm=10^−4^×LDW/LAI_Total_	Cm
CNC, LAI_Total_	Cab=26×LNC (Yang *et al.*, 2015) where LNC=CNC/LAI_Total_	Cab
CNC, LAI_Total_	Car=0.216×Cab (Yang *et al.*, 2015)	Car
/	Fixed Cant to 0	Cant
/	Fixed Cbrown to 0	Cbrown
/	Fixed psoil to 1	psoil
LAI_Total_	LAI=LAI_Total_	LAI
/	Fixed ALA to 50°	ALA
LAI_Total_	hspot=a/LAI_Total_ (Verhoef and Bach, 2003) (a is an empirical parameter and is set as 0.5)	hspot
L, DOY	cos(SZA)=sin(L)sin(δ)+cos(L)cos(δ)cos(h) given δ=23.45sin[360365(284+DOY)] h=15(AST−12)	SZA
/	Fixed VZA to 0	VZA
L, DOY	RAA=SAA–VAA given VAA=0 (when VZA=0) sin(SAA)=cos(δ)sin(h)cos(90°−SZA)	RAA

‘/’ denotes no output for equivalent input; SLA (cm^2^ mg^−1^), specific leaf area, that is leaf area per unit leaf dry weight; LAI_Total_ (m^2^ m^−2^), total leaf area index; LDW (g m^−2^), leaf dry weight per unit planting area; Z_s_, decimal Zadok score for the growth stage; f_dead_, the fraction of dead leaves; LAI_Dead_ (m^2^ m^−2^), leaf area index of senesced or dead leaves; LNC (g m^−2^), leaf nitrogen content per unit leaf area; CNC (g m^−2^), canopy nitrogen content per unit planting area; L (°), local latitude; δ (°), solar declination angle; DOY, day of year; h (°), hour angle; AST (h), apparent solar time for phenotyping crops and here is set at three levels: 10.00, 12.00, and 14.00 h.

### The synthetic datasets

Four sites were chosen to represent diverse soil and climate environments across the Australian wheatbelt (Table 3). Simulations were run with historical weather records from 2000 to 2019, typical soil conditions with best initial soil water in the long term, and local management practices (i.e. fertilization, Table 3; Chenu *et al.*, 2013). For each site in each year, nine cultivars varying in maturity type (Young, spring/very-fast; Gauntlet, spring/fast; Ellison, spring/mid-fast; Wills, spring/mid; Lancer, spring/slow; Forrest, spring/very-slow; Longsword, winter/fast; Kittyhawk, winter/mid; and Manning, winter/slow) and three sowing dates (1 May, 29 May, and 26 June) were selected to characterize different wheat growth patterns (Table 3). In total, 2160 seasons were simulated by APSIM. Daily outputs of major crop traits (Table 2) were sampled fortnightly from emergence to physical maturity, resulting in 25 201 unique records; these APSIM output records were converted into PROSAIL input records using the variable transformation formulas in Table 2, which were used to determine the range and density distribution of each PROSAIL input variable.

**Table 3:** Information of genotype, environment, and management used for simulation to represent the Australian wheatbelt

Cropping area	Site	Latitude	Longitude	Soil classification	PAWC at sowing (mm)	Nitrogen (kg ha^−1^)
West	Merredin	–31.48	118.28	Shallow loamy duplex	86	20-20-30
South-east	Yanco	–34.61	146.42	Brown sodosol	191	40-40-40
East	Narrabri	–30.34	149.76	Grey vertosol	218	130-0-0
East	Gatton	–27.54	152.33	Black vertosol	225	20-30-0

Plant-available water content (PAWC) at sowing is indicated for each soil at its best level of initial soil water (referring to Chenu *et al.*, 2013). Applied nitrogen is represented by ‘x-y-z’: x, applied at sowing; y, z, applied at stage of ‘beginning of stem elongation’ and ‘flag leaf just visible’, respectively.

The synthetic dataset was a set of data pairs constructed from PROSAIL input parameters and corresponding canopy reflectance. Based on the combination mode of PROSAIL input parameters, the synthetic datasets were assigned two names: rdata (combined randomly) and cdata (combined based on constraints). The rdata synthetic dataset was generated in the traditional way used in previous model inversion studies (e.g. Atzberger, 2004; Baret *et al.*, 2007), which used the ranges and distributions of the input parameters converted from APSIM outputs but allowed PROSAIL to be run using samples from full parameter space for any combination of inputs. The second synthetic dataset with biological constraints (cdata) used only input data converted from APSIM output variables to explore a subspace of input parameters (i.e. limited by the APSIM biology) to run PROSAIL. Both rdata and cdata have 25 201 samples. Taking rdata as a table with fixed row–column order, the rdata can be considered as breaking the potential associations among parameters within rows (Supplementary Fig. S1).

The main difference between rdata and cdata is that the value of input parameters can be combined randomly in rdata, while in cdata the input parameter combinations are constrained to wheat growth patterns estimated using APSIM. In fact, some traits (e.g. LAI, Cab, Cm, and Cw) of crops are directly or indirectly associated with each other, meaning that they are not independent and cannot be randomly or arbitrarily combined. For example, the total leaf area gradually increases and reaches its maximum value at the flag leaf during the wheat-growing season; the total leaf water content reduces along with more senescent leaves (Supplementary Fig. S2). This means that the rdata, generated in a random combination way without consideration of biological constraints among traits, may include those ‘unrealistic’ combinations that cannot exist in field conditions.

### The design of simulation experiments

The major objective of these simulation experiments is to compare the theoretical estimation accuracy of target crop traits (i.e. LAI, Cab, Cm, and Cw) based on two synthetic datasets without (rdata) and with (cdata) biological constraints in terms of application of two observation geometries, five wavelength ranges, and three retrieval methods. Reflectance in a specific wavelength range and angles related to observation geometry were used as predictive variables of the predictive model to estimate crop traits with varying retrieval methods. In total, 60 simulation experiments were conducted (2 × 2×5 × 3=60, see below for details).

Because the canopy reflectance is co-determined by the canopy status as well as the observation geometry (i.e. VZA, SZA, and RAA), some scholars considered taking these angles together with reflectance as predictive variables to increase estimation accuracy (e.g. Bacour *et al.*, 2006; Baret *et al.*, 2007; Dhakar *et al.*, 2019). The solar zenith and azimuth angles change during the compositing period of images from a drone during a flight, and the variance of angles within a flight increases with increasing flight time, but calculation of these angles is tedious. Thus, two ways of angle utilization (i.e. ‘wa’, with angles in model input; ‘na’, without angles in model input) were considered here to analyse the effects on estimation accuracy of target crop traits. In this research, these angles only included SZA and RAA as a nadir view was assumed here (VZA=0).

**Fig. 2.**
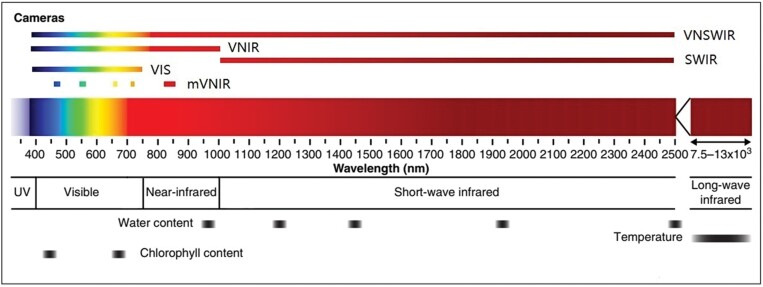
Selected wavelength ranges in the visible to infrared range. There are a few strong absorption peaks at wavelength around 970, 1200, 1450, 1930, and 2500 nm for water (Ma *et al.*, 2019), and around 450 nm and 680 nm for chlorophyll (Féret *et al.*, 2017).

Five sets of wavelength ranges were considered to suit different types of instrumentations and applications: four sets for narrow-band reflectance at 400–2500 nm [visible to shortwave infrared (VNSWIR)], 400–750 nm (visible; VIS), 1000–2500 nm (SWIR), and 400–1000 nm [visible to near infrared (VNIR)], and one set for broad-band reflectance at 400–900 nm [visible to near infrared (multispectral) (mVNIR)] (Fig. 2). The narrow-band reflectance mimics virtual hyperspectral cameras with very high spectral resolution of 1 nm at defined ranges. The broad-band reflectance mimics a commercial multispectral camera taken as an example (MicaSense Rededge, https://www.micasense.com). Five broad-band reflectances of the multispectral camera were resampled from the simulated 1 nm reflectance values based on the spectral response coefficients (provided by MicaSense; Supplementary Fig. S3) in ranges of 465–485 nm (blue), 550–570 nm (green), 663–673 (red), 820–860 nm (NIR), and 712–722 (red-edge). The band reflectance can be calculated as follows:


$$\begin{array}{l} \rho_{i}=\frac{\overset{\lambda_{i,max}}{\underset{j=\lambda_{i,min}}{\sum }}\varphi_{i,j}\times \rho_{j}}{\overset{\lambda_{i,max}}{\underset{j=\lambda_{i,min}}{\sum }}\varphi_{i,j}} \end{array}$$

where ρ_i_ represents simulated band reflectance for the ith band of the camera; ρ_j_ represents the simulated hyperspectral reflectance for the jth wavelength; φ_i,j_ represents the response coefficient of the ith band of the camera for the jth wavelength; and λ_i,min_ and λ_i,max_ represent the lower and upper band wavelength limits for the ith band, respectively.

Three retrieval methods were selected, namely look-up table (LUT), random forest (RF), and feed-forward neural network (FFNN) as described below.

### The implementation of retrieval methods

Based on different combinations of two synthetic datasets, five wavelength ranges, and two types of angle utilization considered, there are 20 (2 × 5×2=20) different simulation experiments that need to be conducted with each retrieval method (Table 4). Experiments 1–10 and 11–20 are constructed from the same subset of rdata and cdata, respectively, with predictive variables varying in wavelength range and angle utilization. For example, there are 353 predictive variables (351 narrow-band reflectance variables at 400–750 nm plus two angle variables including SZA and RAA) for Experiment 1, while Experiment 10 has five predictive variables (five broad-band reflectance variables, i.e. blue, green, red, NIR, and red-edge). The response variables for all experiments are the four crop traits: LAI, Cab, Cm, and Cw.

**Table 4:** Combinations of wavelength range, angle utilization, and synthetic dataset used in simulation experiments based on look-up table, random forest, or feedforward neural network

No.	Model input		Dataset type	No.	Model input		Dataset type
	Wavelength range	Angle utilization			Wavelength range	Angle utilization	
1	VNSWIR	wa	rdata	11	VNSWIR	wa	cdata
2	VIS	wa	rdata	12	VIS	wa	cdata
3	SWIR	wa	rdata	13	SWIR	wa	cdata
4	VNIR	wa	rdata	14	VNIR	wa	cdata
5	mVNIR	wa	rdata	15	mVNIR	wa	cdata
6	VNSWIR	na	rdata	16	VNSWIR	na	cdata
7	VIS	na	rdata	17	VIS	na	cdata
8	SWIR	na	rdata	18	SWIR	na	cdata
9	VNIR	na	rdata	19	VNIR	na	cdata
10	mVNIR	na	rdata	20	mVNIR	na	cdata

Model input represents the predictive variables of look-up table, random forest model, or feedforward neural network model. Angle utilization: ‘wa’, with angles; ‘na’ without angles.

Both predictive variables and response variables were normalized with the zero-mean normalization approach to prevent any scaling issue. No noise or bias was added to synthetic data. For each experiment, 75% of samples of the entire dataset were randomly selected as the training set to generate the LUT or train RF and FFNN models. The remaining 25% of samples were used as the test set to evaluate the theoretical performance of estimation accuracy. A 4-fold cross-validation was conducted with similar results in cross-validation (Supplementary Fig. S4).

#### Look-up table

The LUT is a statistical method based on global searching which was generated here with the training set. The estimation of target variables is achieved by minimizing a cost function that measures the difference in predictive variables between the tested sample (from the test set) and records in the LUT. A cost function based on root mean square error (RMSE) was used to find the best solutions of estimated variables:


$$\begin{array}{l} RMSE=\frac{1}{m}\underset{i=1}{\overset{m}{\sum }}\;{\left(V_{i}-V_{i,LUT}\right)}^{2} \end{array}$$

where m represents the number of predictive variables (here it is reflectance or angles); V_i_ represents the ith predictive variable of a sample from the test set, while V_i, LUT_ represents the ith predictive variable of a record stored in LUT. For estimation of each test sample, the entire LUT was sorted based on RMSE, and the mean of the target traits corresponding to the best solutions leading to minimum RMSE were considered as the final solution of estimated variables (i.e. LAI, Cab, Cm, and Cw). Referring to the setting of the number of best solutions (*k*) in previous studies (e.g. Combal *et al.*, 2003; Quan *et al.*, 2017), we used the mean of the best 10 solutions (*k*=10) instead of the best one solution (*k*=1) to alleviate the ill-posed problem. Increasing *k* from 1 to 10 apparently improved the estimation accuracy (Supplementary Fig. S5) but increasing *k* from 10 to 50 produced no further improvement (data not shown), which was consistent with reported results for *k* selection in Quan *et al.* (2017).

#### Random forest

RF is an ensemble method based on decision trees, belonging to the scope of machine learning. The final prediction is the mean of predictions of multiple decision trees (base learners), trained on different subsets of the same training dataset, in order to overcome the overfitting by an individual base learner (Breiman, 2001). The literature suggests three key hyperparameters (e.g. Lu and He, 2019; Shah *et al.*, 2019), namely the number of trees or base learners (n_estimators), the number of features to consider in finding the best split (max_features), and the minimum number of samples required at a leaf node (min_samples_leaf). max_features was set as log2(n_features) where n_features represents the number of predictive variables to add the attribute disturbance and increase the interindividual differences of base learners (Breiman, 2001).

Several values were evaluated to find the optimal settings, namely, 5, 50, 100, 200, 300, 400, 500, and 1000 for n_estimators, and 1, 5, 10, and 15 for min_samples_leaf. The mean squared error was used for evaluation of model performance during training. n_estimators and min_samples_leaf were set to 200 and 1, respectively, through independent evaluation for each experiment, which provided the optimal performance over the corresponding validation set (i.e. a subset of the training set) for all experiments. The RF model was implemented in Python Version 3.7.2 using the scikit-learn 0.24.2 (https://scikit-learn.org).

#### Feed-forward neural network

FFNNs, also called deep feedforward networks, or multilayer perceptrons, are the quintessential deep learning models (Goodfellow *et al.*, 2016). An FFNN consists of an input layer, an output layer, and one or more hidden layers. According to the universal approximation theorem (Hornik *et al.*, 1989), an FFNN can approximate an arbitrary function even with only one hidden layer that is sufficiently wide. However, simply increasing the number of neurons can easily lead to overparameterization, and previous studies showed that increasing hidden layers typically resulted in better generalization (e.g. Lin *et al.*, 2014; Zhang *et al.*, 2017). Five key hyperparameters (governing the training process and the architecture) need to be determined before training an FFNN, namely the activation function (transform data), the optimizer (specify how the training proceeds), the learning rate (control the move magnitude of weights update in training), the unit number, and the hidden layer number.

To find the optimal model structure, three common values of learning rate (i.e. 0.1, 0.01, and 0.001), 10 activation functions, eight optimizers, five levels of hidden layer number from shallow (1) to deep (5), and eight levels of unit number from narrow (2^4^) to wide (2^11^) were tested. The mean squared error was used as the loss function for evaluation of model performance during training and the maximum training epoch was up to 1000 with an early stopping strategy to prevent overfitting. The configurations of the five parameters (i.e. with 256 units in each of three hidden layers, 0.001 as learning rate, ‘relu’ as activation, ‘Adamax’ as optimizer) were obtained through independent evaluation for each experiment, which provided the optimal and robust performance over the corresponding validation set (i.e. a subset of the training set) for all experiments. The FFNN model was implemented using Keras in TensorFlow 2.3.0 (https://www.tensorflow.org/).

### Validation using the field experiment

In order to demonstrate the applicability of adding biological constraints to synthetic datasets in practice, the final predictive models were tested on the spectral imagery from a field experiment. Due to lack of *in situ* reference data, only models trained on synthetic datasets in the range of the mVNIR were validated on real multispectral images to predict LAI, Cm, and Cw.

**Fig. 3.**
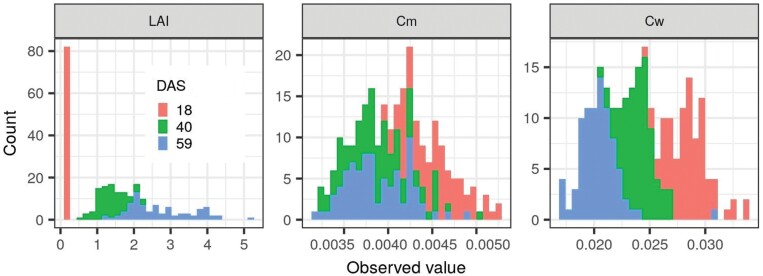
Distribution of observed values of crop traits (LAI, Cm, and Cw) at three UAV phenotyping dates corresponding to tillering stage, start of stem elongation stage, and flag leaf stage, respectively (i.e. 18, 40, and 59 d after sowing).

A wheat experiment was conducted at Gatton, Queensland (27.55°S, 152.33°E) in 2016, and unmanned aerial vehicle (UAV)-based phenotyping was undertaken along with field measurements (for more details of this experiment, refer to Chen *et al.*, 2022). Different genotypes, irrigation regimes, and fertilization regimes created contrasting canopy structures. The whole field was split into four treatment blocks based on irrigation and fertilization regimes, and each block was split into small plots of ~14 m^2^ (2 × 7 m), each with seven rows and a 25 cm row spacing. Quadrat harvests comprising 0.5 m length of four inner rows (~0.5 m^2^) were taken in 84 plots (comprising seven genotypes, four water–nitrogen treatments, and three replicates) to conduct ground measurements of LAI, Cm, and Cw. However, quadrat harvest and UAV phenotyping did not always occur on the same day. Missing LAI measurements from quadrat harvest were interpolated with a fitted piecewise function based on all observed LAIs from quadrat harvests across the growth season (Chen *et al.*, 2022), while missing Cm and Cw values were interpolated with a linear function. The distributions of crop traits at each UAV phenotyping date between plant emergence and flag leaf are presented in Fig. 3.

The multispectral camera used in this study was a MicaSense RedEdge camera (https://www.micasense.com). The multispectral data were captured from a UAV-based phenotyping platform during 10.00 h to 14.00 h on three defined dates (Fig. 3), with a flight height of 20 m resulting in a ground sample distance of ~1.3 cm. Raw multispectral images were processed in Pix4Dmapper software (https://www.pix4d.com/product/pix4dmapper-photogrammetry-software) to generate the calibrated reflectance of each band for the whole field. A normalized difference vegetation index (NDVI) map was computed from red and NIR bands based on the formula: NDVI=(NIR–Red)/(NIR+Red). The NDVI map was used to generate the vegetation–background binary map based on threshold classification. The threshold was empirically set as 0.5 (tillering stage), 0.65 (stem elongation stage), and 0.75 (flag leaf stage). These threshold values can effectively classify soil pixels as background and avoid classifying green leaves as background, especially when plants are small (Supplementary Fig. S6). Using the binary map as a mask, the value of background pixels in the original reflectance map was replaced with the corresponding soil reflectance used in synthetic data, resulting in a new reflectance map named ‘background-corrected reflectance map’. Each background-corrected reflectance map for the entire field was then segmented into individual plots according to the experimental design. Marginal areas from adjacent plots and plot gaps as well as harvest areas were trimmed from the plot images. The pixel-scale reflectance from the background-corrected reflectance map was averaged to generate the plot-scale reflectance that was used in predictive models to predict crop traits. Finally, the predicted plot value for LAI, Cm, and Cw was compared with observed LAI, Cm, and Cw, respectively, to calculate the related metrics for performance evaluation. Further details about image processing can be found in the study of Chen *et al.* (2022).

## Results and discussion

In the synthetic data study, compared with models using rdata, the models using cdata significantly improved the estimation accuracy of four response variables or crop traits (i.e. LAI, Cab, Cm, and Cw), although the magnitude of improvement depended on the application of angle utilization, wavelength ranges, and retrieval methods (Supplementary Fig. S7). The theoretical effects of these three factors are discussed in detail below. Additionally, models trained on synthetic datasets in the range of mVNIR were tested on real multispectral data to predict LAI, Cm, and Cw.

### Slight effects of utilization of angles related to observation geometry

**Fig. 4.**
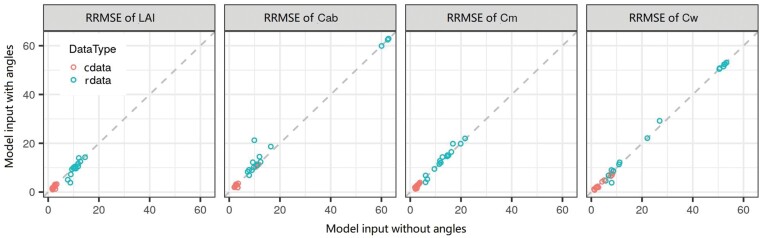
Comparison of relative root mean squared error (RRMSE, %) of four crop traits (i.e. LAI, Cab, Cm, and Cw) predicted on synthetic test datasets with different retrieval methods and wavelength ranges with or without angles as model input. cdata and rdata denote the synthetic dataset with and without biological constraints, respectively. Both data types had 30 independent data points, 10 for each method with various model inputs (Table 4).

The relative root mean squared error (RRMSE) was compared for four crop traits (i.e. LAI, Cab, Cm, and Cw) predicted for models with and without angles (i.e. SZA and RAA) related to observation geometry as model inputs (Table 4). Utilization of observation geometry in the models only caused slight changes of RRMSE to estimate crop traits (i.e. –4.9% to 1.9%, –0.9% to 11.3%, –2.0% to 3%, and –4.2% to 2.3% for LAI, Cab, Cm, and Cw, respectively, in the test dataset), whichever retrieval method and wavelength range was used (Fig. 4). Changes in RRMSE ranged from –4.9% to 11.3% in the models trained on rdata and from –1.5% to 0.5% in the models trained on cdata. The minor improvements in the latter were caused by the better performance of models trained on cdata (see below for details). The utilization of observation geometry resulted in slight improvements of estimation accuracy when retrieving with FFNN but no improvements with LUT or RF (Supplementary Fig. S7), which agreed with methodologies in the literature; for example, these angles have been used in neural network-based methods (e.g. Bacour *et al.*, 2006; Dhakar *et al.*, 2019), but rarely used in non-neural network-based methods including LUT and RF (e.g. Shah *et al.*, 2019; Upreti *et al.*, 2019). In this context, it was concluded that it would be reasonable to omit these angles from model inputs in the subsequent analyses.

### FFNN outperformed RF and LUT in joint estimation of multiple crop traits

**Fig. 5.**
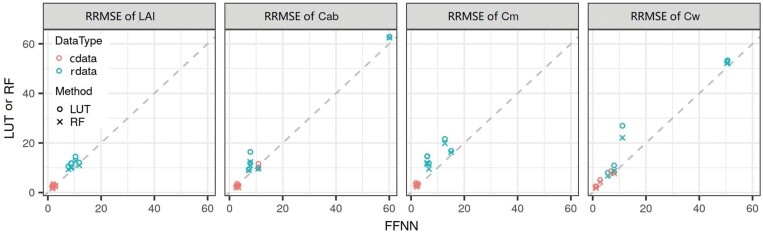
Comparison of relative root mean squared error (RRMSE, %) of four crop traits (i.e. LAI, Cab, Cm, and Cw) predicted on synthetic test datasets with feedforward neural network (FFNN) and look-up table (LUT) or random forest (RF). cdata and rdata denote the synthetic dataset with and without biological constraints, respectively. Both data types had 10 independent data points: five for FFNN versus LUT and the other five for FFNN versus RF (without angles in model input, Table 4).

Compared with LUT and RF, on average, the use of FFNN reduced RRMSE by 1.9% (LAI), 2.7% (Cab), 5.5% (Cm), and 4.2% (Cw) for rdata and within 1% for the four traits for cdata (Fig. 5). The better performance from FFNN is thought to be due to superior performance of neural networks in approximating the function between inputs and outputs and optimizing the mathematical constraints among multiple outputs, given the use of a universal approximation theorem (Hornik *et al.*, 1989). The estimation of crop traits for rdata was mainly based on the correlation between reflectance and traits, while the mathematical constraints, learnt during FFNN training, provided supplemental information to improve estimation accuracy. This finding was consistent with previous studies reporting that multitask deep learning models can exploit correlations among traits to improve prediction accuracy for green area index and the fraction of absorbed photosynthetically active radiation (e.g. Danner *et al.*, 2021) as well as physiological traits related to photosynthesis (Furbank *et al.*, 2021). The information carried in reflectance across the entire range from 400 nm to 2500 nm was insufficient to predict Cm but sufficient to predict LAI, Cab, and Cw based on sensitivity analysis results (Supplementary Fig. S8). This conclusion was consistent with research for similar parameter ranges (Danner *et al.* 2019), which suggested that the averaged reduction of RRMSE in using FFNN instead of RF or LUT for estimation of Cm was larger than that for LAI, Cab, and Cw. Since biological constraints were more effective than mathematical constraints learnt during training FFNN for joint estimation (see below for details), the use of cdata appeared to weaken the superiority of FFNN.

### Significant effects of chosen subsets of wavelength ranges

**Fig. 6.**
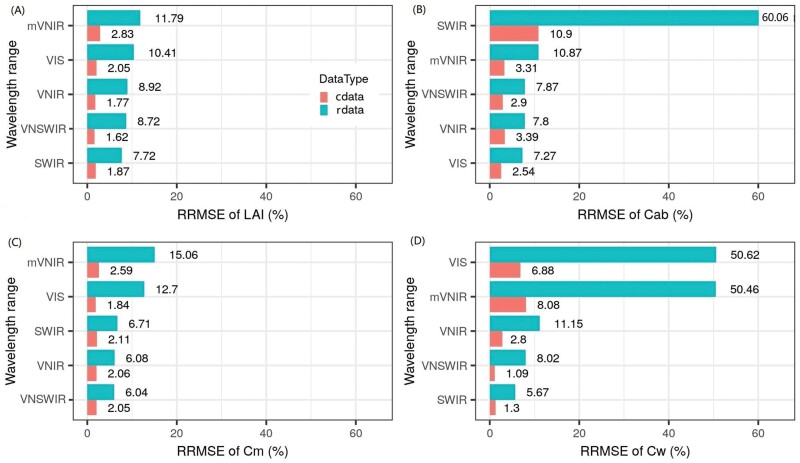
Relative root mean squared error (RRMSE, %) of LAI (A), Cab (B), Cm (C), and Cw (D) predicted on synthetic test datasets with feedforward neural network (FFNN) from reflectance in varying wavelength ranges (i.e. VIR, VIS, SWIR, VNIR, and mVNIR; Fig. 2). RRMSEs were order based on values from rdata. cdata and rdata denote the synthetic dataset with and without biological constraints, respectively.

The effective wavelength ranges for prediction varied for crop traits. For estimation of LAI with rdata, the RRMSE for each wavelength range was ordered as: SWIR (7.7%)<VNSWIR (8.7%)≈VNIR (8.9%)<VIS (10.4%)<mVNIR (11.8%) (Fig. 6A). SWIR was not suitable to retrieve Cab for rdata (RRMSE=60.1%), while the RRMSE was ordered as: VIS (7.3%)<VNIR (7.8%)≈VNSWIR (7.9%)<mVNIR (10.9%) for the other four ranges (Fig. 6B). The RRMSE of Cm estimation for rdata was smaller in VNSWIR/VNIR/SWIR (6.0–6.7%) than in VIS (12.7%) and mVNIR (15.1%) (Fig. 6C). The best wavelength range for Cw estimation for rdata was SWIR (5.7%), followed by VNSWIR (8.0%) and VNIR (11.2%), while mVNIR and VIS were not suitable (RRMSE>50%) (Fig. 6D). The suitability of wavelength range for prediction of specific crop traits was supported by sensitivity analysis results (Supplementary Fig. S4) and confirmed with previous reported results (e.g. Baret *et al.*, 1998; Gitelson, 2004; Ustin *et al.*, 2009; Féret *et al.*, 2017).

Compared with models trained on rdata, the models trained on cdata significantly improved estimation accuracy, resulting in substantial reduction of RRMSE by 5.9–9.0% for LAI, 4.4–49.2% for Cab, 4.0–12.5% for Cm, and 4.4–43.7% for Cw (Fig. 6). However, for models trained on cdata, the SWIR, VNSWIR, and VNIR models still resulted in smaller RRMSE to VIS or mVNIR models for estimation of LAI, Cm, and Cw, while VIS, VNIR, VNSWIR, and mVNIR models resulted in smaller RRMSE than the SWIR model for estimation of Cab.

**Fig. 7.**
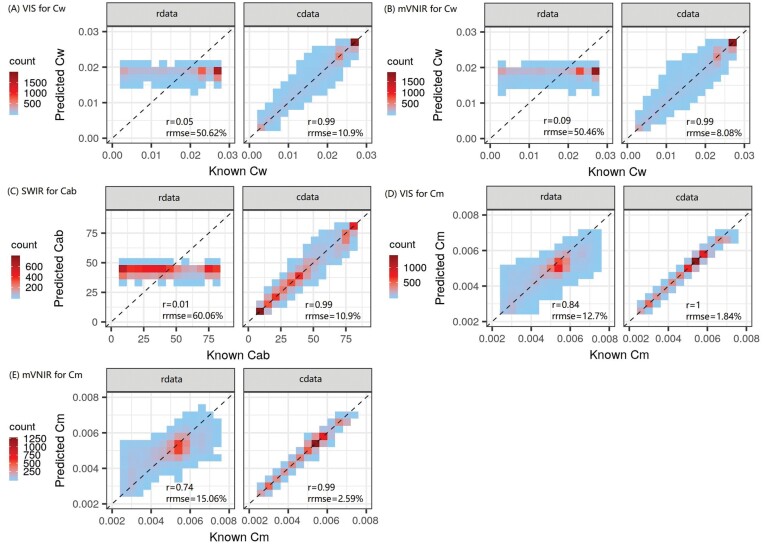
Known values and values of crop traits predicted with the feedforward neural network (FFNN) over the synthetic test dataset. Leaf water content (Cw, g cm^−2^) was predicted from VIS (A) and mVNIR (B); leaf chlorophyll content (Cab, µg cm^−2^) was predicted from SWIR (C); leaf dry matter content (Cm, g cm^−2^) was predicted from VIS (D) and mVNIR (E). See Fig. 2 for details of wavelengths. cdata and rdata denote the synthetic dataset with and without biological constraints, respectively. Raw data have been aggregated to 15 bins based on the full range of *x*- and *y*-axes. Completed results predicted with LUT, RF, and FFNN from varying wavelength ranges for four crop traits (i.e. LAI, Cab, Cm, and Cw) can be found in Supplementary Figs S9, S10, and S11, respectively.

The models trained on cdata also resulted in relatively accurate estimation of a specific crop trait from a given wavelength range which was unsuitable for estimation of the corresponding crop trait for rdata. For example, models using cdata enabled Cw to be retrieved from VIS (RRMSE=6.9%) and mVNIR (RRMSE=8.1%) (Fig. 7A, B), enabled Cab to be retrieved from SWIR (RRMSE=10.9%) (Fig. 7C), and significantly improved Cm estimation from VIS (RRMSE=1.8%) and mVNIR (RRMSE=2.6%) (Fig. 7D, E). Under these conditions, the estimation of Cw, Cab, or Cm was mainly based on prior biological constraints between crop traits defined in APSIM instead of the correlations between crop traits and radiometric information. Overall, the models trained on cdata could achieve accurate joint estimation in VNIR, with an RRMSE of 1.8, 3.4, 2.1, and 2.8%, respectively, for LAI, Cab, Cm, and Cw (Fig. 6).

### Application of the FFNN model on real UAV-based multispectral images

**Fig. 8.**
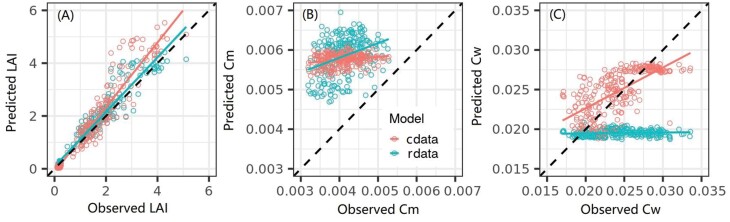
Observed values of leaf area index (LAI, m^2^ m^−2^) (A), leaf dry matter content (Cm, g cm^−2^) (B), and leaf water content (Cw, g cm^−2^) (C) against their estimated values predicted with feedforward neural network (FFNN) models over real multispectral images captured from a UAV-based phenotyping platform. The two FFNN models were trained over synthetic datasets without (rdata) and with (cdata) biological constraints, respectively. All statistical metrics are summarized in Table 5. Validation for LUT and RF models can be found in Supplementary Fig. S12.

The established FFNN models trained over synthetic datasets in the range of mVNIR were applied on MicaSense multispectral images captured from a UAV-based phenotyping platform to evaluate models’ practical performance with ground references of crop traits (i.e. LAI, Cm, and Cw) between the plant emergence and flag leaf stages(Fig. 8; Table 5). In the comparison of observed values against predictions, the FFNN model trained on rdata showed a good agreement for LAI (RMSE=0.36 m^2^ m^−2^); however, the model trained on cdata resulted in less accurate estimation of LAI (RMSE=0.54 m^2^ m^−2^), with an increasing overestimation for higher observed LAI (Fig. 8A; Table 5). The model trained on rdata failed to predict Cw from multispectral data, with a near horizontal slope of 0.01 between predictions and observations (Fig. 8C; Table 5), due to the lack of effective spectral information in the range of mVNIR for Cw prediction as mentioned above. In contrast, the model trained on cdata enable Cw to be predicted with a slope of 0.51 (Fig. 8C; Table 5), in which situation the prediction was based on prior biological constraints between crop traits expressed in cdata. No matter whether the models were trained on rdata or cdata, poor estimation of Cm (Fig. 8B) occurred, with systematic overestimation to leverage the estimation of other traits, as the estimation of Cm was mainly based on its correlation with other traits described above. Despite the fact that practical performance was less accurate than the theoretical performance due to uncertainties in simulations and measurements, the model trained on cdata achieved good estimation of LAI (RMSE=0.54 m^2^ m^−2^) and Cw (RMSE=0.0028 g cm^−2^) on real multispectral data (Table 5).

**Table 5:** Theoretical (testing over synthetic data) and practical performance (testing over experimental data) of feed-forward neural network (FFNN) models trained over a synthetic dataset

Test set	Model	Crop trait	*r*	RMSE	RRMSE	Regression function
Synthetic data (*n*=6298)	rdata	LAI	0.99	0.17	11.79	*y*=0.98×*x*+0.04
	cdata	LAI	1	0.04	2.83	*y*=*x*
	rdata	Cm	0.74	0.00084	15.06	*y*=0.44×*x*+0.003
	cdata	Cm	0.99	0.00013	2.59	*y*=0.99×x
	rdata	Cw	0.64	0.0093	50.46	*y*=0.0049×*x*+0.019
	cdata	Cw	0.95	0.0015	8.08	*y*=0.98×*x*+0.001
Experimental data (*n*=252)	rdata	LAI	0.96	0.36	24.99	*y*=1.03×x+0.09
	**cdata**	**LAI**	**0.96**	**0.54**	**37.94**	***y*=1.22×*x*+0.11**
	rdata	Cm	0.35	0.0018	44.55	*y*=0.37×*x*+0.001
	**cdata**	**Cm**	**0.23**	**0.0017**	**41.91**	***y*=0.08×*x*+0.01**
	rdata	Cw	0.10	0.0060	24.80	*y*=0.01×*x*+0.02
	**cdata**	**Cw**	**0.69**	**0.0028**	**11.34**	***y*=0.51×*x*+0.01**

Evaluation metrics include correlation coefficient (r), root mean squared error (RMSE), relative root mean squared error (RRMSE), and regression function. ‘cdata’ and ‘rdata’ represents the synthetic dataset with and without biological constraints, respectively. The unit of RMSE for leaf area index (LAI), leaf dry matter content (Cm), and leaf water content (Cw) is m^2^ m^−2^, g cm^−2^, and g cm^−2^, respectively. For the regression function, ‘*x*’ and ‘*y*’ in the regression function represent the known or observed value and its prediction, respectively.

The practical performance of the FFNN model trained over cdata is formatted in bold.

### Potentials and limitations

The estimation accuracy of crop traits via deep learning models was substantially improved when biological constraints were introduced to the synthetic dataset as the inference/estimation of crop traits was not solely based on correlations between crop traits and radiometric information, but was also based on biological constraints among simulated crop traits. Crop traits are not independent but are explicitly or implicitly associated with each other, so they cannot be randomly combined during sampling (Supplementary Fig. S1). The ill-posed problem was effectively alleviated by the introduction of biological constraints in two ways. (i) A higher quality training dataset was provided. Those combinations of parameter values that were unrealistic to crop growth patterns defined in APSIM were omitted from the entire parameter space, resulting in a more realistic subset (i.e. cdata). This higher quality training dataset resulted in more appropriate solutions to be found in searching LUT and a better approximation function between predictive and response variables in training of RF and FFNN. (ii) More effective constraints among response variables (i.e. crop traits) were learned in FFNN training. Given the fully connected nature of FFNN, the constraints among response variables would be learned to reduce possible combinations of values of response variables corresponding to given predictive variables. The constraints were totally mathematically based for rdata but biologically based for cdata such that crop traits within training data are constrained to APSIM’s biophysical model of trait interactions. Our study indicated that these biological constraints were able to reduce the dependence on effective information carried in spectra (Figs 6, 7) to achieve accurate estimation of crop traits. The FFNN model trained on cdata enabled the prediction of leaf water content (Cw) from real multispectral images in the visible to NIR range (i.e. mVNIR) (Table 5).

The accurate estimation presented in the synthetic data was obtained based on assumptions and hypotheses (resulting in optimistic situations rather than actual cases for variable retrieval) and might not be achieved from experimental data due to model and measurement uncertainties (Table 5). In this research, we fixed the ALA (average leaf angle) to an overall mean value for generating synthetic datasets as it is difficult to determine ALA accurately, which conflicts with the fact/knowledge that the variable ALA was reported to significantly affect canopy reflectance (e.g. Verrelst and Rivera, 2017; Danner *et al.*, 2019). LAI and ALA are not independent during the inversion process. Assigning a fixed value to ALA can stabilize the inversion process in circumstance where constraints are not clear (Jacquemoud *et al.*, 1995); that is probably the reason why ALA was fixed in some studies (e.g. Thorp *et al.*, 2012; Guo *et al.*, 2019). The relationships for leaf mesophyll structure parameter (Ns) and specific leaf area (SLA) and for Cab and leaf nitrogen content (LNC) in this study were found under specific situations and they are not so consistent across varying environmental or/and genotypic conditions. However, the models trained on cdata effectively avoided unreasonable solutions being found during LUT searching or RF and FFNN training, resulting in improvement of prediction accuracy (Supplementary Figs S8–S10). Despite the limiting situations represented in the experimental dataset, in the best situation, models trained on experimental data generally achieved more accurate estimation of crop traits than those trained on synthetic data (without biological constraints) when testing on the same subset of experimental data (Camacho *et al.*, 2021), which supports the effectiveness of biological constraints that are implicitly included in experimental data.

The major limitation factor is the gap between observed and simulated spectra under given conditions due to simplification in RTMs, when the predictive model, completely trained on a synthetic dataset, is applied to retrieve crop traits from experimental data in practice (e.g. Danner *et al.*, 2019). Considering crop growth patterns, the synthetic dataset with biological constraints should better represent the actual situations than that without biological constraints. Previous studies provided evidence that a model completely trained on a synthetic dataset (without biological constraints) could achieve acceptable estimation accuracy of crop traits (e.g. LAI, Cab, etc.) on experimental data (e.g. Dhakar *et al.*, 2019; Upreti *et al.*, 2019). For example, Danner *et al* (2021) reported that an FFNN model trained over synthetic data (without constraints, with artificial noise) was able to predict crop traits of multiple crops from 242 hyperspectral reflectance bands in the range of VNSWIR with an RRMSE of 9% (LAI) and 5% (Cab) on synthetic test data (our study: 9% for LAI and 8% for Cab in VNSWIR with 2101 bands) and of 28% (LAI) and 19% (Cab) on experimental data. It is reasonable to expect that a model trained on a novel synthetic dataset with biological constraints will result in satisfactory estimation on experimental data. The estimation accuracy of Cab was reported to be improved by using a matrix-based LUT to consider the relationship between LAI and Cab, reducing RMSE from 10.5–22.9 μg cm^−2^ to 7.9–13.8 μg cm^−2^ (M. Xu *et al.*, 2019). Different from the study of M. Xu *et al.* (2019), the framework of integrating APSIM and PROSAIL proposed here demonstrated a more systematic way to add biological constraints throughout the growing season and across environments. However, there is no guarantee that the model trained on cdata can always outperform the model trained on rdata when it is used on experimental data (Supplementary Fig. S12). The likely reason is that the biological constraints, defined in APSIM, may not sufficiently describe the actual situations of wheat development and growth, and errors in the characterization of environment and genotype. Hence, we suggest testing this proposed concept framework on real datasets captured with varying sensors from more diverse environments. Additionally, for a specific application in practice, the APSIM model can be calibrated with phenology and biomass information in the conventional way before being used to inform PROSAIL input parameters. This calibrated APSIM and PROSAIL should be able to generate improved synthetic data with reflectance spectra highly correlated to the real ones captured in the field, which in turn will result in more accurate estimation of crop traits predicted with models trained over the improved synthetic data.

Fine hyperspectral reflectance at 1 nm spectral resolution theoretically provided large capacity to retrieve crop traits depending on the range of wavelength (Fig. 6). Some commercial systems already provide fine hyperspectral reflectance, such as Compact Airborne Spectrographic Imager (408-947@7.5 nm, imaging sensor) (e.g. Haboudane *et al.*, 2004), Cubert FireflEYE V185 (450-950 nm@4 nm, imaging sensor) (e.g. Zhu *et al.*, 2019), and ASD FieldSpec 4 Hi_Res (350-2500 nm@1 nm) (e.g. Shah *et al.*, 2019, point sensor). However, fine reflectance is still hard and expensive to obtain from current commercial sensors. We also mimicked a common multispectral camera with five broad-band reflectance in blue, green, red, NIR, and red-edge regions (i.e. mVNIR). The use of mVNIR could also reveal advantages of using a deep learning model (i.e. FFNN) and adding biological constraints (i.e. cdata) to retrieve crop traits from reflectance in theory (Fig. 6) and in practice (Fig. 8). Regardless of uncertainties/unrealities of physical models and possible error propagation in variable transformation between models, superior solutions can be expected in the future as these models (i.e. PROSAIL andAPSIM) have been evolving to better describe the reality (e.g. Verhoef, 1984; Jacquemoud and Baret, 1990; Féret *et al.*, 2017; Holzworth *et al.*, 2014, 2018), and their validity for addressing practical problems (e.g. crop trait retrieval or crop growth simulation) has been widely reported (e.g. Jacquemoud *et al.*, 2009; Chenu *et al.*, 2017; Berger *et al.*, 2018). As it is foreseeable that many innovative applications can be achieved from the integration of models, this will further stimulate the improvement of current versions towards more powerful models in the future.

### Conclusion

This research demonstrated a conceptual framework of integrating APSIM and PROSAIL to predict crop traits, and the advantages of adding biological constraints in synthetic training data for joint estimation of multiple crop traits (i.e. LAI, Cab, Cm, and Cw). Our research indicated that angles related to observation geometry had slight effects on estimation accuracy, and FFNN achieved more accurate results in estimation of multiple crop traits than LUT and RF. Adding biological constraints among related variables significantly improved the estimation accuracy of crop traits. The models trained on a synthetic dataset with biological constraints enabled the estimation of crop traits not only based on correlations between crop traits and radiometric information, but also based on associations among crop traits themselves, which allowed Cab and Cw to be estimated from reflectance in SWIR and VIS or mVNIR, respectively. Although the good estimations presented in theory might not be completely reproduced in practical applications due to measurement and model uncertainties, the validation of an established FFNN model on real UAV-based multispectral images in the mVNIR range suggested the effectiveness of adding biological constraints in training synthetic datasets for prediction of multiple crop traits.

## Supplementary data

The following supplementary data are available at *JXB* online.

Protocol S1. APSIM–PROSAIL coupling model.

Fig. S1. Concept map of combinations of PROSAIL input parameters.

Fig. S2. Measured and predicted leaf water content for total leaves.

Fig. S3. Spectral response coefficient for each band of the MicaSense RedEdge camera.

Fig. S4. Cross-validation results for LUT.

Fig.S5. Comparison of the number of best solutions on LUT predictions.

Fig. S6. Schematics of NDVI threshold classification results.

Fig. S7. Estimation accuracy from LUT, RF, or FFNN on the synthetic test dataset.

Fig. S8. Global sensitivity analysis.

Fig. S9. Known values against predictions with LUT on the synthetic test dataset.

Fig. S10. Known values against predictions with RF on the synthetic test dataset.

Fig. S11. Known values against predictions with FFNN on the synthetic test dataset.

Fig. S12. Observed values against predictions with LUT, RF, and FFNN on real data.

erac291_suppl_supplementary_protocol_S1_figures_S1-S12

## Data Availability

The detailed description for the proposed concept framework to integrate APSIM and PROSAIL can be found in the supplementary data, and the iterative protocols are deposited at protocols.io: https://www.protocols.io/workspaces/integration-of-apsim-and-prosail/. Details (https://www.apsim.info/apsim-next-generation/) and source code (https://github.com/APSIMInitiative/ApsimX) of the APSIM Next Generation model used in this study are openly available online. Details and source code of the PROSAIL model used in this study are openly available at http://teledetection.ipgp.jussieu.fr/prosail/. Other data and source codes supporting this work are available from the corresponding author upon request.

## Abbreviations

Cab: leaf Chl *a* and *b* content per leaf area
Cant: leaf anthocyanin content per leaf area
Car: leaf carotenoid content per leaf area
Cbrown: leaf brown pigment concentration
cdata: synthetic dataset with biological constraints of which PROSAIL input parameters are constrained to the crop growth pattern defined in APSIM
CGM: crop growth model
Cm: leaf dry matter content per leaf area
Cw: leaf water content per leaf area or leaf equivalent water thickness
FFNN: feedforward neural network
hspot: hot spot size parameter
LAI: leaf area index
LIDF: leaf inclination distribution function
LUT: look-up table
mVNIR: visible to near infrared (multispectral)
Ns: leaf mesophyll structure parameter
*r*: Pearson correlation coefficient
RAA: relative azimuth angle
rdata: synthetic dataset without biological constraints of which PROSAIL input parameters can be combined randomly
RF: random forest
RMSE: root mean squared error
RRMSE: relative root mean squared error
rsoil: reflectance of soil as a libertarian surface
RTM: radiative transfer model
SWIR: shortwave infrared
SZA: solar zenith angle
UAV: unmanned aerial vehicle
VIS: visible
VNIR: visible to near infrared
VNSWIR: visible to shortwave infrared
VZA: viewing zenith angle.
