# Supplementary material for: Integrating a crop growth model and radiative transfer model to improve estimation of crop traits based on deep learning
Source: J Exp Bot. 2022 Jun 30;73(19):6558–74. doi: 10.1093/jxb/erac291 (PMC9629788; doi:10.1093/jxb/erac291)
Supplement: erac291_suppl_supplementary_protocol_S1_figures_S1-S12 [file erac291_suppl_supplementary_protocol_s1_figures_s1-s12.pdf]

Integrating crop growth model and radiative transfer model to improve estimation of crop traits based on deep learning

Qiaomin Chen[1, 2, *], Bangyou Zheng[2], Tong Chen[3], Scott C. Chapman[1, *]

SUPPLEMENTARY DATA

Protocol S1 APSIM-PROSAIL coupling model

The APSIM-PROSAIL coupling model is established by passing output variables of APSIM to PROSAIL as input variables. This permits APSIM-PROSAIL to estimate canopy reflectance from 400 to 2500 nm in 1 nm interval on daily step given that required parameters are specified. The output LAI of APSIM-NG can be directly passed to PROSAIL as input variable. The leaf brown pigment parameter (C_{brown}) and leaf anthocyanins content (C_{ant}) is not considered here and set as zero (C_{brown}=0, C_{ant}=0) through all growth stages. The transformation of other variables is based on a series of equations below.

The leaf structure parameter (Ns) can be calculated from specific leaf area (SLA, cm^2 mg^{-1}) according to the description in (Jacquemoud and Baret, 1990), with a maximum allowance to 2.5 (Berger et al., 2018).

$$Ns = (0.9 * SLA + 0.025)/(SLA - 0.1) \tag{1}$$

$$SLA = 10 * \mathrm{LAI}_{Total}/LDW \tag{2}$$

where LAI_{Total} (m^2 m^{-2}) represents the total leaf area index; LDW (g m^{-2}) represents the leaf dry weight per unit planting area.

The leaf dry matter content per leaf area (C_m, g cm^{-2}) can be calculated from LDW and LAI_{Total}, as shown in Eq (3).

$$C_m = 10^{-4} * LDW/\mathrm{LAI}_{Total} \tag{3}$$

Previous studies have reported there is a strong linear relationship between leaf nitrogen content (LNC, g m^{-2}), leaf chlorophyll content (C_{ab}, µg cm^{-2}) and leaf carotenoid content (C_{ar}, µg cm^{-2}) (Jacquemoud et al., 1996; Yang et al., 2015). For wheat, C_{ab} and C_{ar} can be calculated using Eq (4) and Eq (5), respectively.

$$C_{ab} = 26 * LNC \tag{4}$$

$$C_{ar} = 0.216 * C_{ab} \tag{5}$$

$$LNC = CNC/LAI_{Total} \tag{6}$$

where CNC (g m^{-2}) represents canopy nitrogen content per unit planting area.

Leaf equivalent water thickness (C_w, cm) is an equivalence of leaf water content per leaf area (g cm^{-2}), referring to the water mass per unit leaf area. It is calculated with Eq (7).

$$C_w = (TLFW - TLDW)/TLA \quad (7)$$

where TLFW (g) is the total leaf fresh weight; TLDW (g) is the total leaf dry weight; TLA (cm^2) is the total leaf area. Leaf water content is co-impacted by growth stage, soil water status and weather conditions. For a field experiment conducted in 2016 at the experimental station of Gatton Campus, the University of Queensland (27.57°S, 152.33°E) as described in Hu et al. (2019), observations appear to show that leaf water content was highly correlated with growth stages or fraction of dead leaves at canopy level regardless of genotype and irrigation-nitrogen application (Fig. S2). Therefore, here C_w was approximately calculated using Eq (8).

$$C_w = (1 - f_{dead})C_w^{green} + f_{dead}C_w^{fead} \quad (8)$$

$$C_w^{green} = -0.000196Z_s + 0.0298 \quad (9)$$

$$C_w^{dead} = 0.0157exp(-2.54f_{dead}) \quad (10)$$

where Z_s is the decimal zadok score for the growth stage (Zadoks et al., 1974). The fraction of dead leaves (f_{dead}) can be expressed as the ratio of leaf area index of senesced or dead leaves (LAI_{Dead}) divided by leaf area index of total leaves (LAI_{Total}).

$$f_{dead} = LAI_{Dead}/LAI_{Total} \quad (11)$$

The hot spot size parameter (hspot=ι/h) is equal to the ratio of the correlation length of leaf projection in the horizontal plane (ι) and the canopy height (h), which can be estimated in two way (Verhoef, 1998). The growth of wheat leaf area is mainly achieved by increasing number of leaves, which is usually accompanied by an increase in plant height, so the hspot parameter for wheat can be calculated as:

$$\text{hspot} = a/LAI_{Total} \quad (12)$$

where a is an empirical parameter and here is set as 0.5, with an allowed maximum of 0.5 for hspot parameter (Dong et al., 2019; Verhoef and Bach, 2003).

Leaf angle, also called leaf inclination angle, is the angle between leaf surface normal and horizontal surface normal. In nature, insertion leaf angles of individual leaf vary from 0 to 90, but average leaf angles often lie between 20 and 70 (Wilson, 1967) and the leaf angle varies across the leaf – closer to vertical at point of insertion to stem, and then closer to horizontal at some point as the leaf blade bends under gravity into a curve with the tip almost always drooping downwards on longer leaves For wheat crops, it is common to have only one or two

leaves left on the plant at late growing stage approaching maturity. In this research, based on our knowledge, ALA is set at its common value (50˚) for the aim of simplification.

The reflectance of soil (r_{soil}) is determined by the geographical location of the crop. In order to simplify the adjustment of soil properties in a continuous fashion, PROSAIL implements a hypothesis that soil reflectance spectrum in general could be represented by mixing a wet soil spectrum and a dry spectrum via adjustment parameter (p_{soil}, ranging from 0 to 1), with an equation for calculation of r_{soil} as: $r_{soi} = r_{soi\_dry} \times p_{soil} + r_{soi\_wet} \times (1-p_{soil})$, where r_{soi_dry} and r_{soi_wet} represents the reflectance of soil under dry and wet conditions, respectively. There is default reflectance spectrum for r_{soi_dry} and r_{soi_wet} provided in PROSAIL, which can be used as alternative if no suitable soil reflectance is available. In this study, the soil reflectance was measured with a ASD FieldSpec Spectroradiometer (https://www.malvernpanalytical.com/asd) at sowing dates before (dry condition) and after (wet condition) irrigation. The surface of soil is dry at most time except right after irrigation or heavy rain event (>5mm). In addition, the influence of soil background can be neglected when LAI>3 according to our sensitivity analysis results and results from (Atzberger et al., 2003) in which situation canopy generally reaches a high ground cover (Ramirez-Garcia et al., 2012). Thus, here in this study it is set as 1 ($p_{soil} = 1$). In addition, the soil reflectance in 400-2500cm can be simulated based on default soil reflectance provided in PROSAIL and actual soil reflectance derived from spectral images with a soil reflectance calibration method proposed in Chen et al. (2022).

The three sun-sensor-geometry parameters (SZA, VZA and RAA expressed in degree) are determined by the physical environment (location, date and time) where images would be captured. The solar zenith angle (SZA) is the angle between the sun's rays and the vertical panel, which is expressed as the equation below.

$$\cos(SZA) = \sin(L)\sin(\delta) + \cos(L)\cos(\delta)\cos(h) \quad (13)$$

$$\delta = 23.45\sin\left(\frac{360}{365}(284 + DOY)\right) \quad (14)$$

$$h = 15(AST - 12) \quad (15)$$

$$AST = LST + ET \pm (SL - LL) - DS \quad (16)$$

$$ET = 9.87\sin(2B) - 7.73\cos(B) - 1.5\sin(B) \quad (17)$$

$$B = \frac{360}{364}(DOY - 81) \quad (18)$$

where L (°) is local latitude (values of L north of the equator are positive and those south ae negative); δ (°) is the solar declination angle; h (°) is the hour angle; DOY is the day of the year;

AST is the apparent solar time (based on the apparent angular motion of the sun across the sky); LST is the local standard time (the time on a clock of a locality); ET (min) is the equation of time; SL (°) and LL (°) is standard and local longitude respectively; DS is the daylight saving (either 0 or 60 min). If a location is east of Greenwich, the sign of Eq. (16) is minus (-), and if it is west, the sign is plus (+). If a daylight-saving time is used, this must be subtracted from the local standard time. The term DS depends on whether daylight saving time is in operation (usually from end of March to end of October) or not. This term is usually ignored from this equation and considered only if the estimation is within the DS period. Here in this research, values of L, DOY and AST are determined by location that crops are growing in and date/time crops are phenotyped. Here L and DOY are set according to APSIM output and, AST is set at 12:00.

The viewing zenith angle (VZA) is the angle between the sensor and the horizontal surface normal where the object is located. It is calculated from coordinates of both sensor and object.

$$\cos{(VZA)} = \frac{\overrightarrow{OZ} * \overrightarrow{OV}}{|\overrightarrow{OZ}| * |\overrightarrow{OV}|} \tag{19}$$

where $\overrightarrow{OZ}$ is a vector and denotes the vertical direction of object; $\overrightarrow{OV}$ is another vector and denotes the direction from object to sensor. Here the viewing zenith angle is set as zero ($VZA = 0$) as the sensor above the object is placed perpendicular to the object's projection on horizontal surface when imaging.

The relative azimuth angle (RAA) is the difference between solar azimuth angle (SAA) and viewing azimuth angle (VAA). The solar azimuth angle (westward is designated as positive) can be calculated with the equation below.

$$\sin{(SAA)} = \frac{\cos(\delta)\sin{(h)}}{\cos{(90° - \mathrm{SZA})}} \tag{20}$$

If cos(h) > tan(δ)/tan(L), this equation is correct; otherwise, it needs to be adjusted and is (|SAA|-90°) for morning hours and (90°-|SAA|) for afternoon hours.

The viewing azimuth angle is the angle between the sensor and the north direction of object on the horizontal surface. It is obtained from sensor's and object's coordinates.

$$\cos{(VAA)} = \frac{\overrightarrow{ON} * \overrightarrow{OV^*}}{|\overrightarrow{ON}| * |\overrightarrow{OV^*}|} \tag{21}$$

where $\overrightarrow{ON}$ is a vector and denotes the north direction of object on the horizontal surface; $\overrightarrow{OV^*}$ is another vector and denotes the direction from object to sensor on the horizontal surface. In this case, the viewing azimuth angle needs to be adjusted by minus 180° (that is VAA-180) in order to matching range of solar azimuth angle. Here the viewing azimuth angle is zero as well because the viewing zenith angle is set as zero as mentioned above. Thus, the relative azimuth angle equals the solar azimuth angle.

Supplementary figures

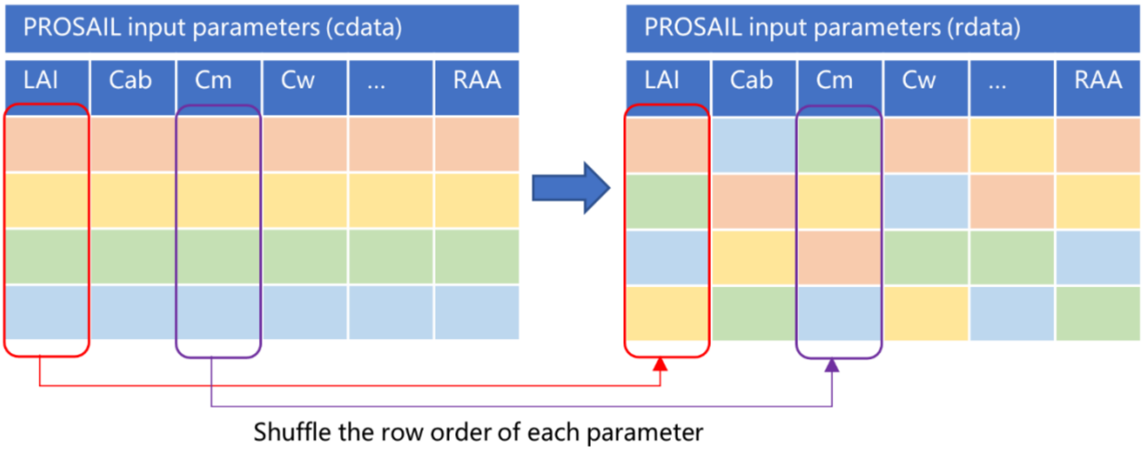

Fig. S1. Concept map of combinations of PROSAIL input parameters in cdata and rdata. The input parameters of cdata in each row are combined based on biological constraints defined in APSIM, so they are coloured in the same colour; while the input parameters of rdata in each row are combined randomly, so they can be coloured in different colours.

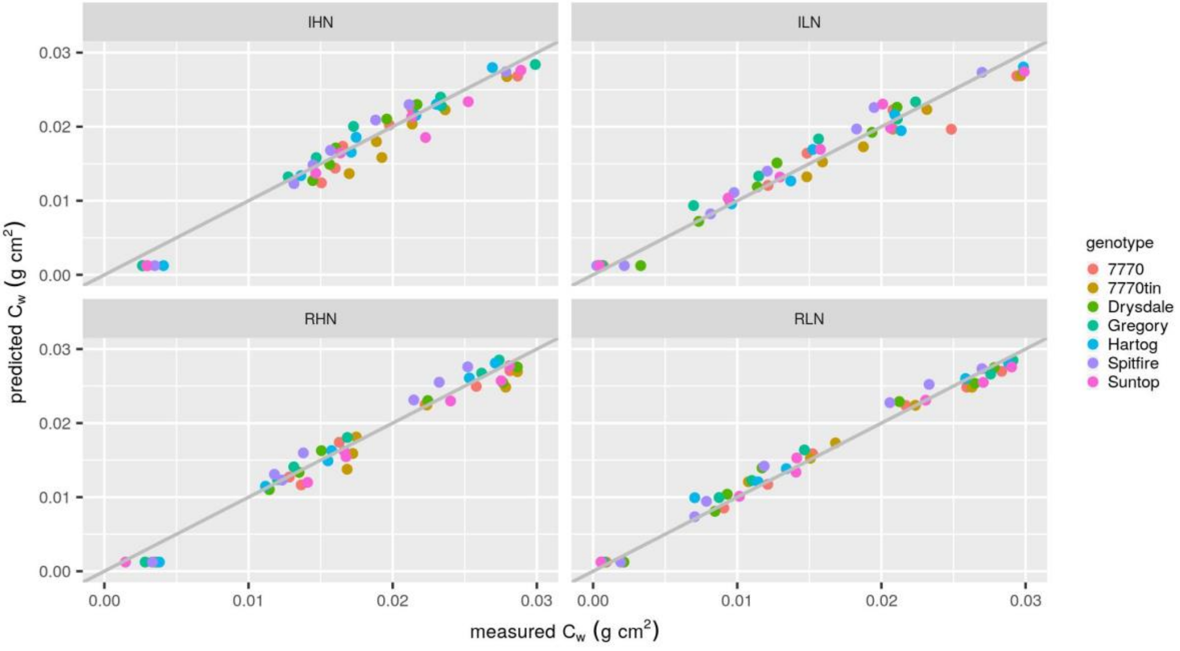

Fig. S2. Measured leaf water content (Cw) for total leaves against its prediction retrieved with proposed approach defined in Eq. (8) of Protocol S1.

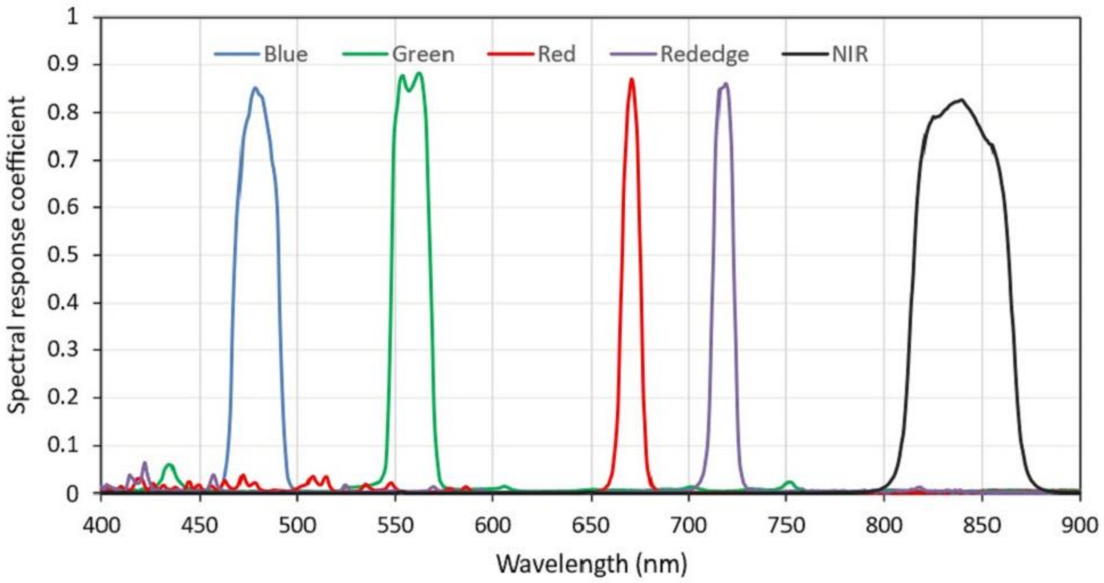

Fig. S3. Spectral response coefficient for each band of MicaSense RedEdge camera. The centre wavelength and bandwidth for each band is as: Blue (475 nm, 20 nm), Green (560 nm, 20 nm), Red (668 nm, 10 nm), Rededge (717 nm, 10 nm), and Near Infrared (NIR: 840 nm, 40 nm).

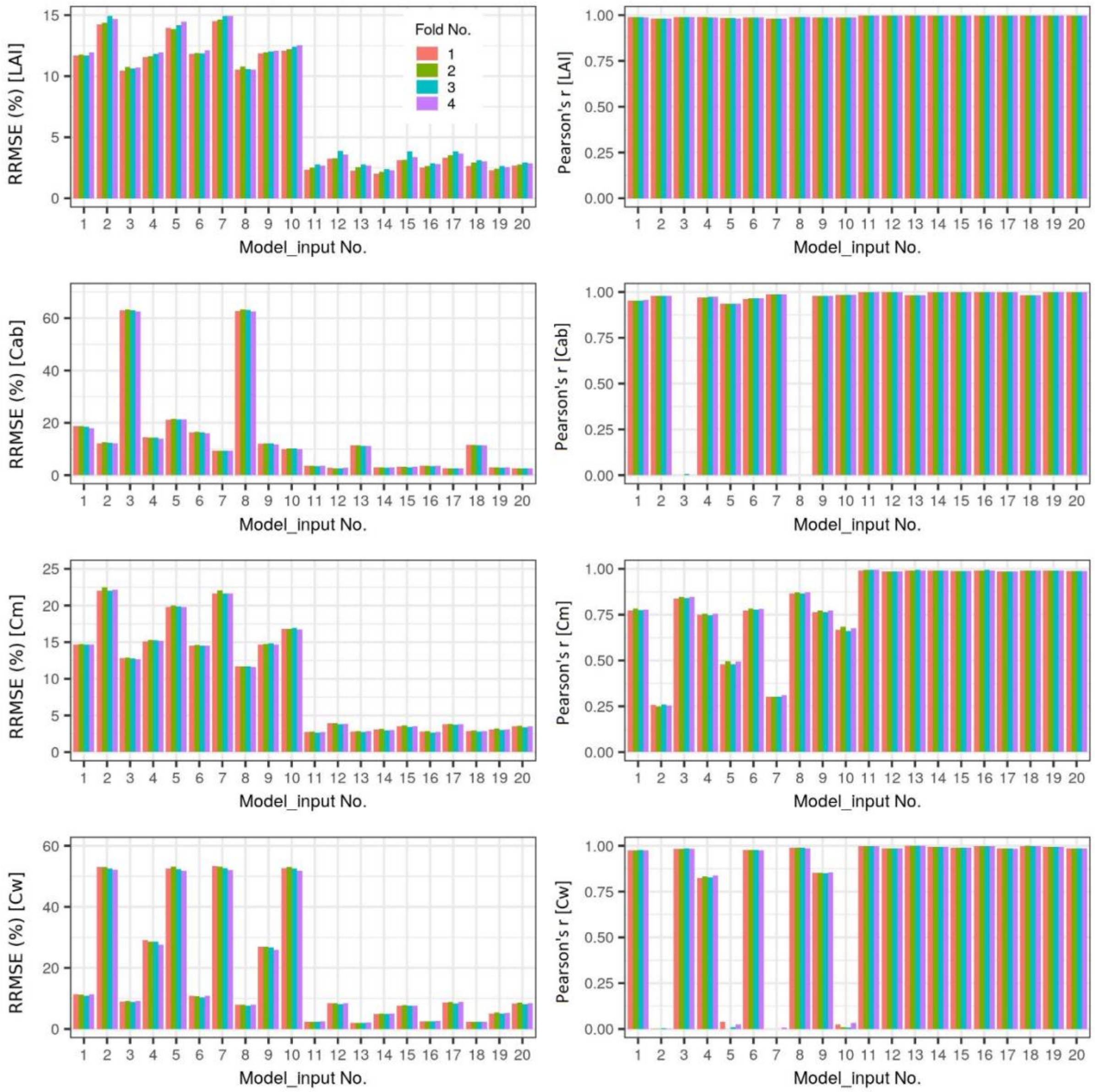

Fig. S4. Relative RMSE (RRMSE, %) and correlation coefficient (Pearson's r) of crop traits over each of the synthetic test dataset for cross validation retrieved with LUT. Fold No. indicates the fold number for the 4-fold cross validation. The structure of model input and data type corresponding to 'Model_input No'. can refer to Table 4. Results of cross validation for RF and FFNN are omitted.

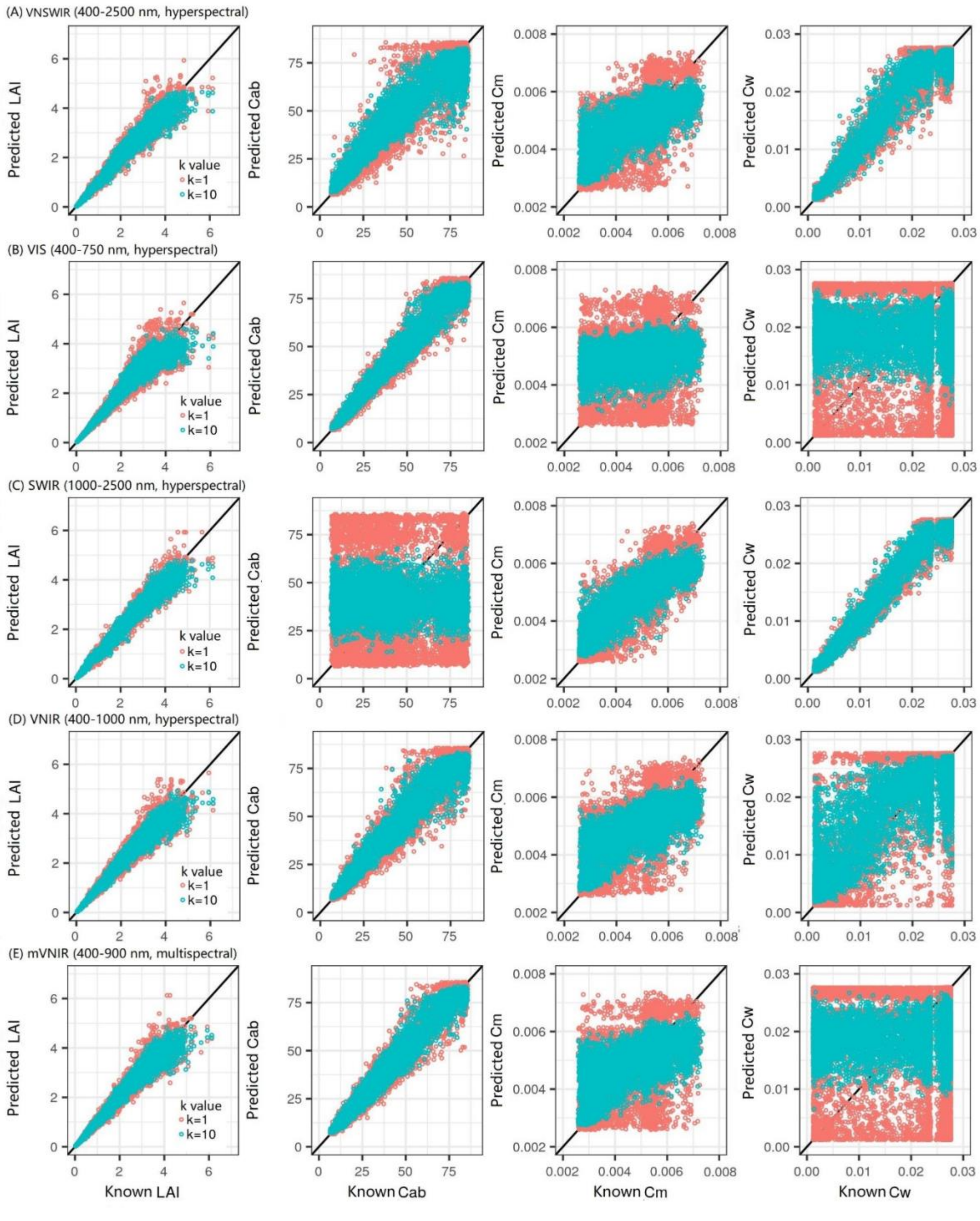

Fig. S5. Known value of crop trait and its estimated value retrieved with look-up table (LUT) over the synthetic test dataset (rdata). LUT input variables only include reflectance in specific wavelength range. ‘k’ indicates the number of best solutions resulted in minimum differences: “k=1” represents the results from the best one solution, while “k=10” represents the results from the means of the best 10 solutions. The synthetic dataset used here is rdata without biological constraints. The unit of LAI, Cab, Cm and Cw is m^2 m^{-2}, μg cm^{-2}, g cm^{-2} and g cm^{-2}, respectively.

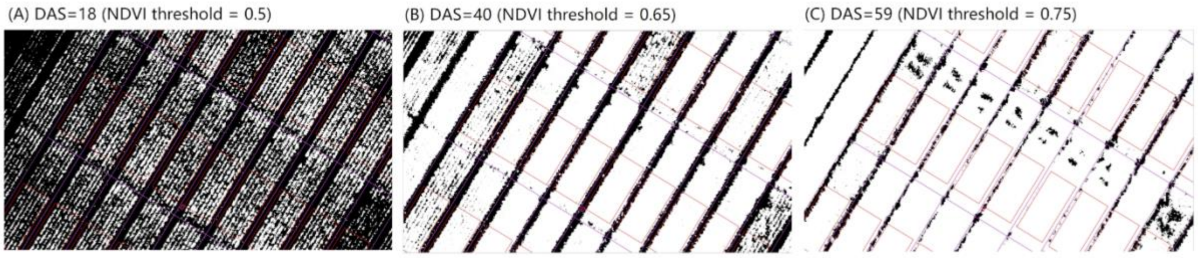

Fig. S6. Schematics of vegetation (white) and background (black) binary map based on NDVI threshold classification for three phenotyping dates at the early stages of wheat growth. Only a few plots under irrigation and high nitrogen treatment (IHN) are chosen here to present the classification results. The purple rectangle represents the full extent of the plot, and the red rectangle inside represents the extent of the trimmed plot.

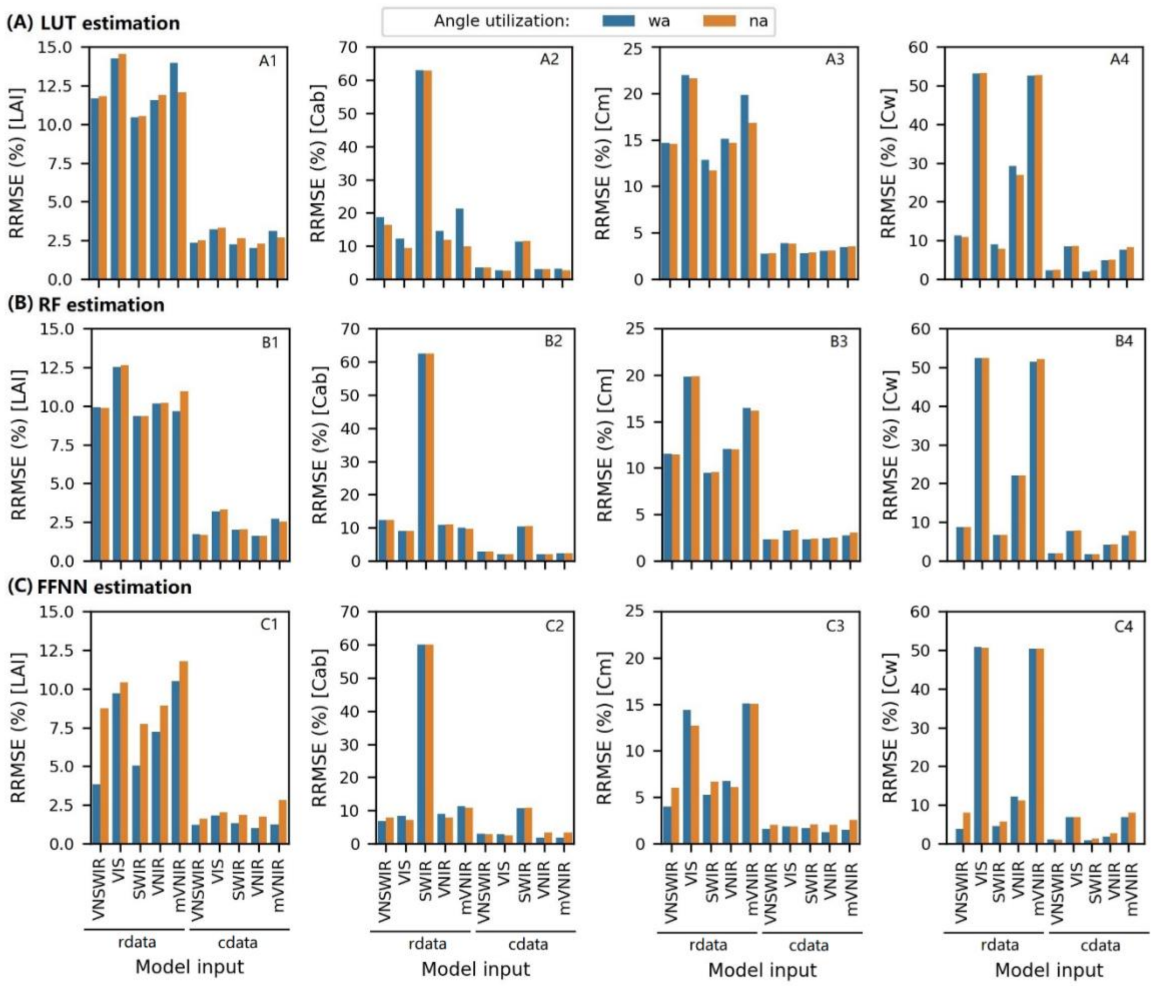

Fig. S7. Relative RMSE (RRMSE, %) of crop traits over the synthetic test dataset retrieved with LUT (A), RF (B) or FFNN (C). Three retrieval methods: LUT, look-up table; RF, random forest; FFNN, feedforward neural network. Synthetic dataset: cdata, with biological constraints; rdata, without biological constraints. Model input variables considered two parts of information - reflectance and angles (Table 4). Angle utilization: “wa”, with angles; “na”, without angles.

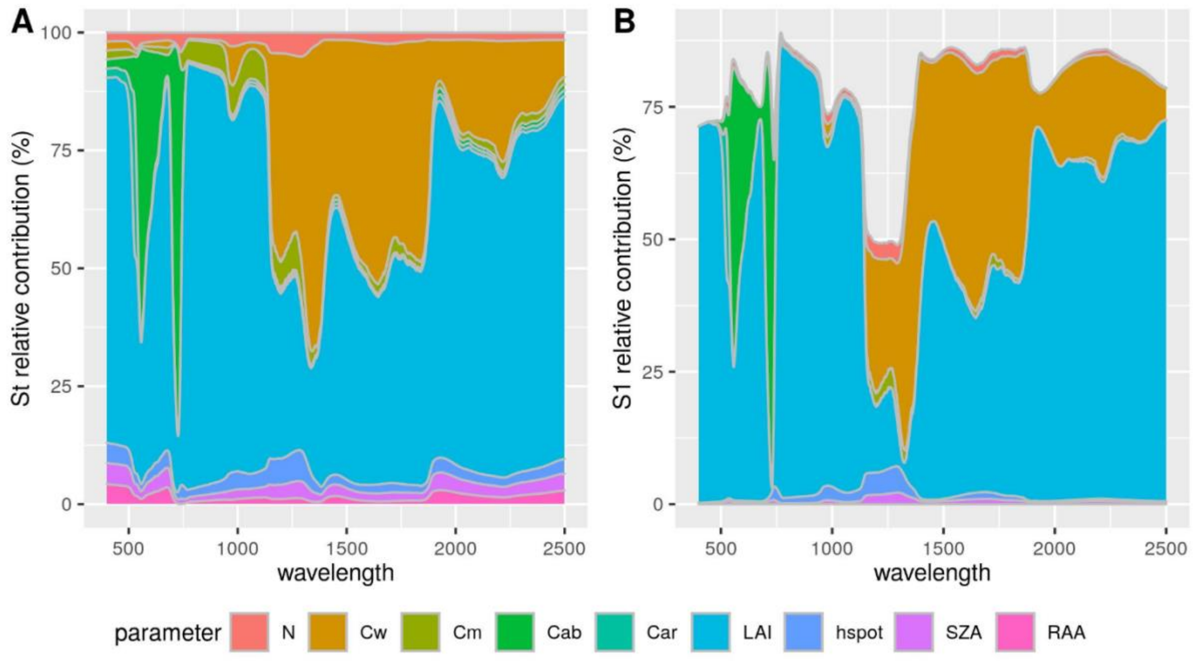

Fig. S8. Relative contribution of total effect (A) and first-order effect (B) for each input parameter on canopy reflectance computed by PROSAIL using EFAST global sensitivity analysis for rdata.

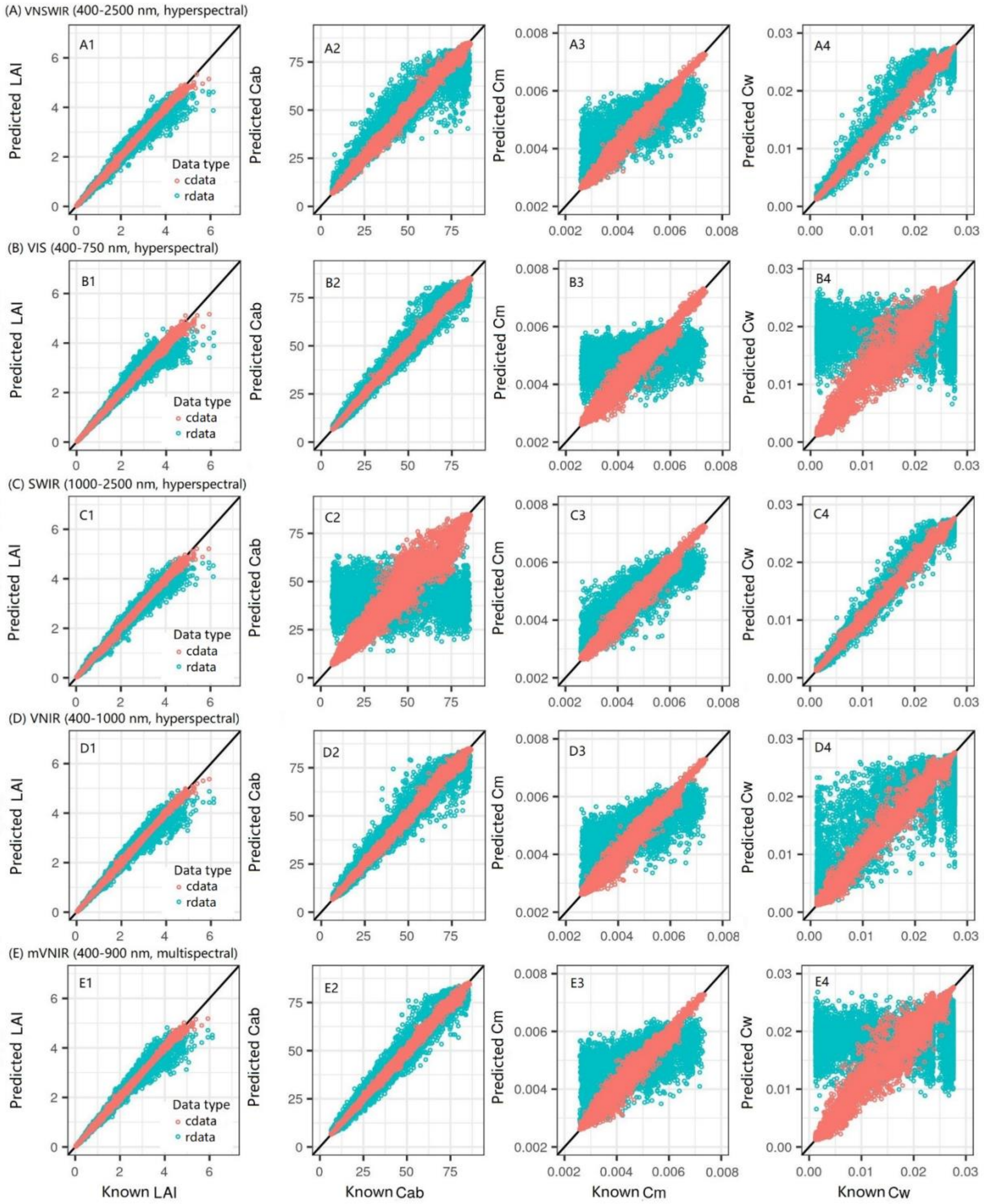

Fig. S9. Known value of crop trait and its estimated value retrieved with look-up table (LUT) models over the synthetic test dataset. LUT inputs only included reflectance in specific wavelength range. cdata and rdata denote the synthetic dataset with and without biological constraints, respectively. The unit of LAI, Cab, Cm and Cw is m^2 m^{-2}, μg cm^{-2}, g cm^{-2} and g cm^{-2}, respectively.

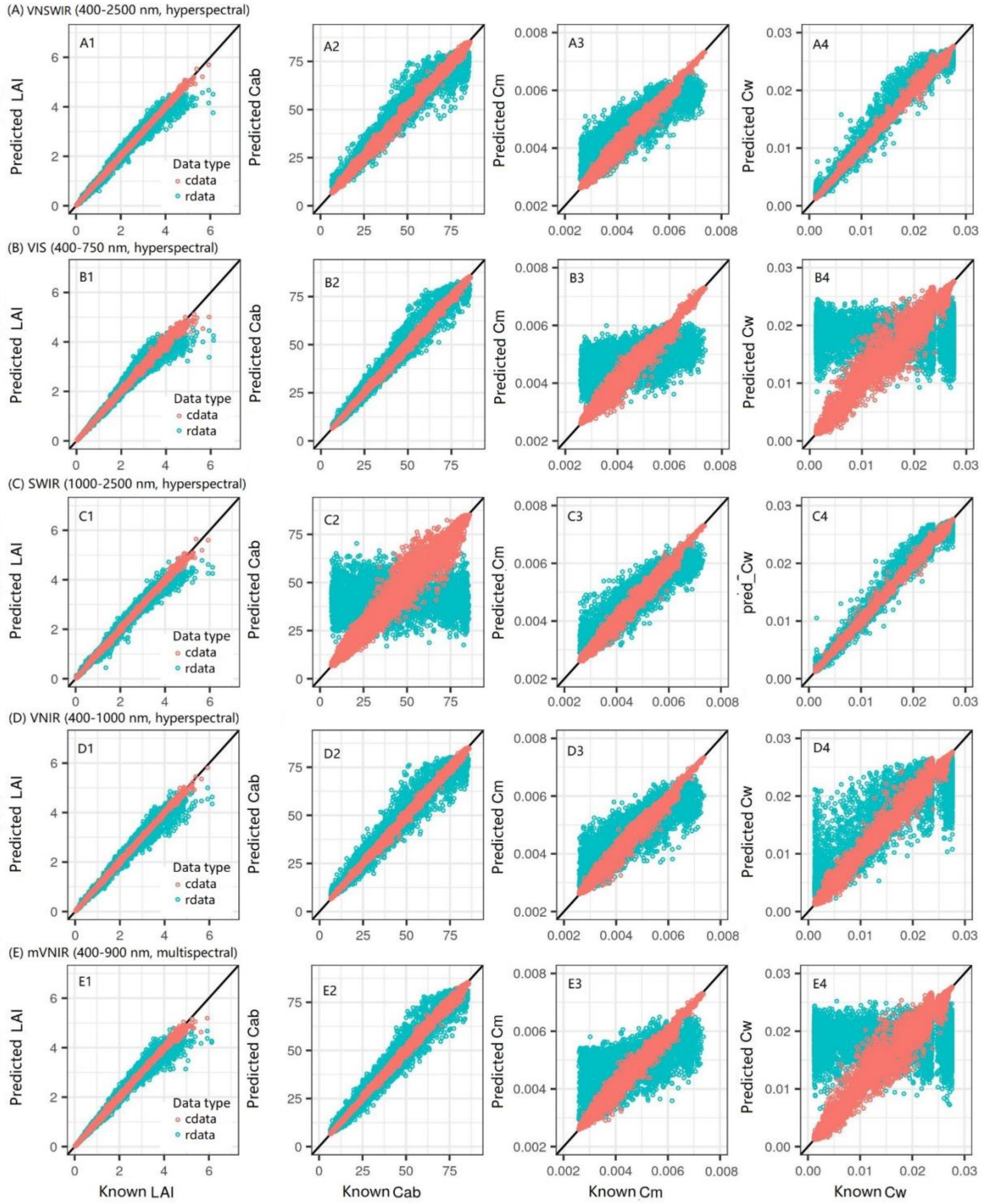

Fig. S10. Known value of crop trait and its estimated value retrieved with random forest (RF) models over the synthetic test dataset. RF inputs only included reflectance in specific wavelength range. cdata and rdata denote the synthetic dataset with and without biological constraints, respectively. The unit of LAI, Cab, Cm and Cw is m^2 m^{-2}, μg cm^{-2}, g cm^{-2} and g cm^{-2}, respectively.

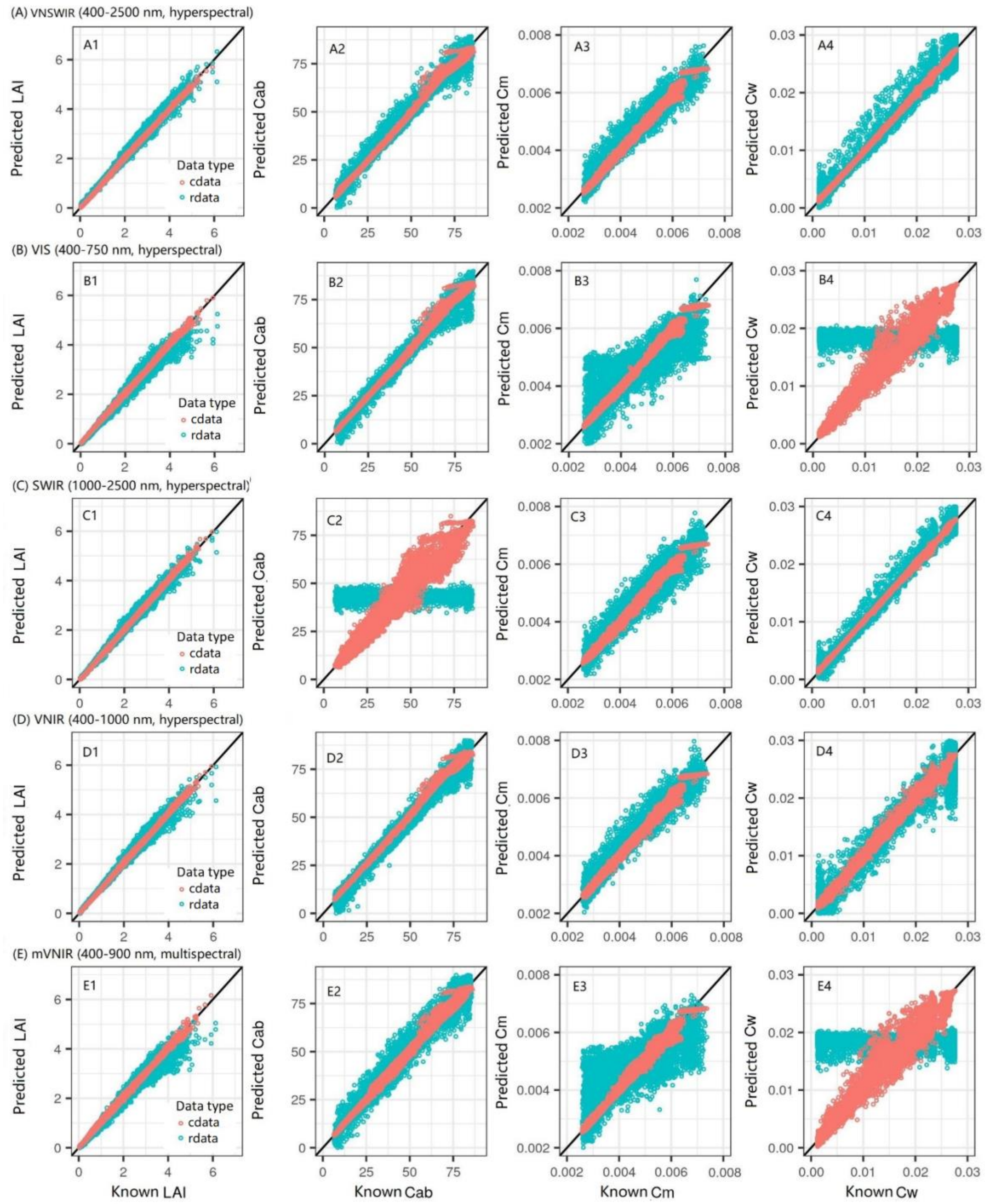

Fig. S11. Known value of crop trait and its estimated value retrieved with feedforward neural network (FFNN) models over the synthetic test dataset. FFNN inputs only included reflectance in specific wavelength range. cdata and rdata denote the synthetic dataset with and without biological constraints, respectively. The unit of LAI, Cab, Cm and Cw is m^2 m^{-2}, µg cm^{-2}, g cm^{-2} and g cm^{-2}, respectively.

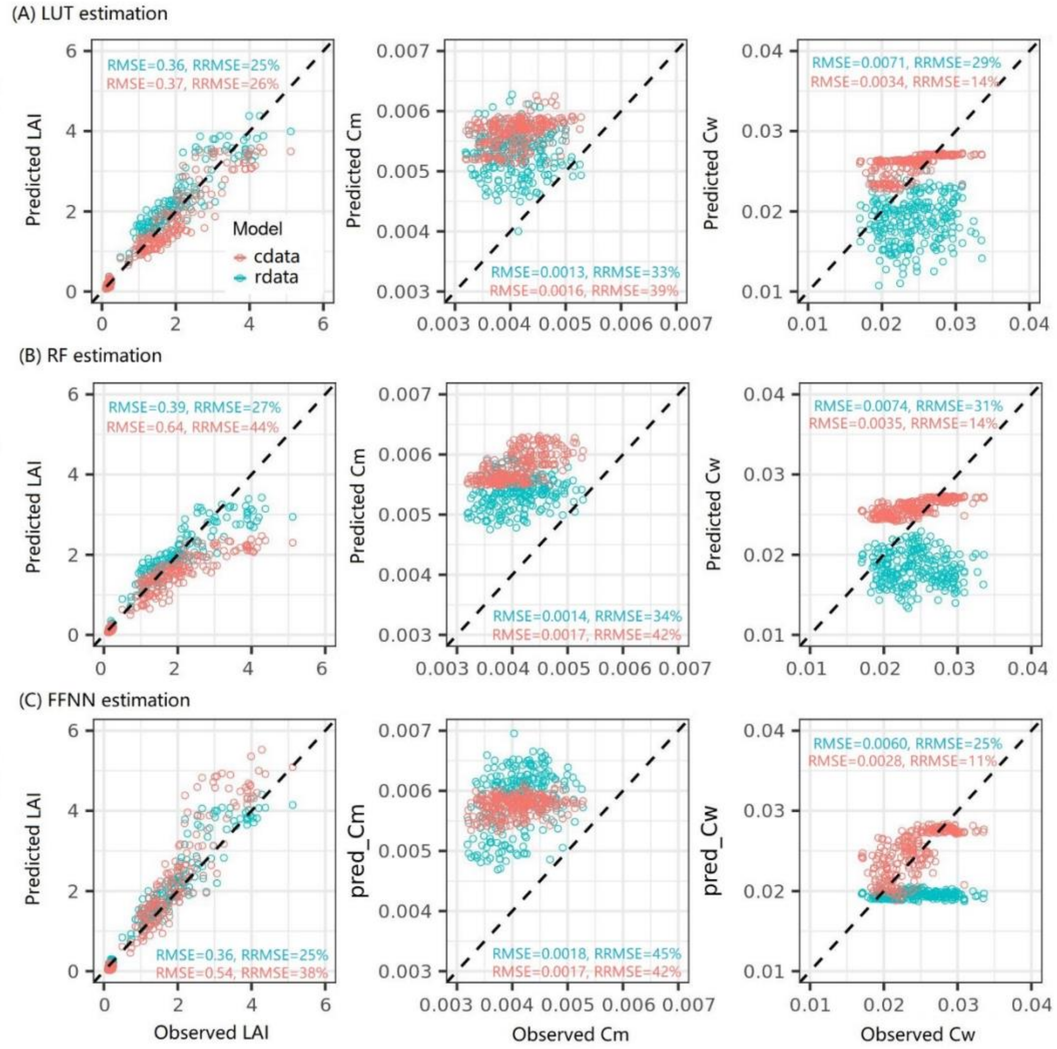

Fig. S12. Observed values of crop traits (LAI, Cm, Cw) against their estimated values predicted with established look-up tables (LUT) (A), random forest (RF) (B) and feedforward neural network (FFNN) (C) models over the same real multi-spectral dataset. In the same panel, the two models were trained over synthetic datasets without (rdata) and with (cdata) biological constraints, respectively.

References

Atzberger C, Jarmer T, Schlerf M, Kötz B, Werner W. 2003. Retrieval of wheat bio-physical attributes from hyperspectral data and SAILH+ PROSPECT radiative transfer model. 3rd EARSeL Workshop on Imaging Spectroscopy. 473–482.

Berger K, Atzberger C, Danner M, D'Urso G, Mauser, W, Vuolo F, Hank T. 2018. Evaluation of the PROSAIL model capabilities for future hyperspectral model environments: A review study. Remote Sensing. 10, 85.

Chen Q, Zheng B, Chenu K, Hu P, Chapman SC. 2022. Unsupervised plot-scale LAI phenotyping via UAV-based imaging, modelling and machine learning. Plant Phenomics (in press).

Dong T, Liu J, Shang J, Qian B, Ma B, Kovacs JM, Walters D, Jiao X, Geng X, Shi Y. 2019. Assessment of red-edge vegetation indices for crop leaf area index estimation. Remote Sensing of Environment. 222, 133–143.

Hu P, Guo W, Chapman SC, Guo Y, Zheng B. 2019. Pixel size of aerial imagery constrains the applications of unmanned aerial vehicle in crop breeding. ISPRS Journal of Photogrammetry and Remote Sensing. 154, 1–9.

Jacquemoud S, Baret F. 1990. PROSPECT: A model of leaf optical properties spectra. Remote Sensing of Environment. 34, 75–91.

Jacquemoud S, Ustin SL, Verdebout J, Schmuck G, Andreoli G, Hosgood B. 1996. Estimating leaf biochemistry using the PROSPECT leaf optical properties model. Remote Sensing of Environment. 56, 194–202.

Ramirez-Garcia J, Almendros P, Quemada M. 2012. Ground cover and leaf area index relationship in a grass, legume and crucifer crop. Plant and Soil Environment. 58, 385–390.

Verhoef W. 1998. Theory of radiative transfer models applied in optical remote sensing of vegetation canopies. Wageningen Agricultural University.

Verhoef W, Bach H. 2003. Simulation of hyperspectral and directional radiance images using coupled biophysical and atmospheric radiative transfer models. Remote Sensing and Environment. 87, 23–41.

Wilson JW. 1967. Stand Structure and Light Penetration. III. Sunlit Foliage Area. Journal of Applied Ecology. 4, 159–165.

Yang G, Zhao C, Pu R, Feng H, Li Z, Li H, Sun C. 2015. Leaf nitrogen spectral reflectance model of winter wheat (Triticum aestivum) based on PROSPECT: simulation and inversion. Journal of Applied Remote Sensing. 9, 095976.

Zadoks JC, Chang TT, Konzak CF. 1974. A decimal code for the growth stages of cereals. Weed Research. 14, 415–421.
